# Transcriptome-based selection and validation of optimal house-keeping genes for skin research in goats (*Capra hircus*)

**DOI:** 10.1186/s12864-020-06912-4

**Published:** 2020-07-18

**Authors:** Jipan Zhang, Chengchen Deng, Jialu Li, Yongju Zhao

**Affiliations:** College of Animal Science and Technology, Southwest University, Chongqing Key Laboratory of Forage & Herbivore, Chongqing Engineering Research Center for Herbivores Resource Protection and Utilization, Chongqing, 400715 P. R. China

**Keywords:** House-keeping genes, Reference genes, Goat, Skin, ComprFinder method

## Abstract

**Background:**

In quantitative real-time polymerase chain reaction (qRT-PCR) experiments, accurate and reliable target gene expression results are dependent on optimal amplification of house-keeping genes (HKGs). RNA-seq technology offers a novel approach to detect new HKGs with improved stability. Goat (*Capra hircus*) is an economically important livestock species and plays an indispensable role in the world animal fiber and meat industry. Unfortunately, uniform and reliable HKGs for skin research have not been identified in goat. Therefore, this study seeks to identify a set of stable HKGs for the skin tissue of *C. hircus* using high-throughput sequencing technology.

**Results:**

Based on the transcriptome dataset of 39 goat skin tissue samples, 8 genes (*SRP68*, *NCBP3*, *RRAGA*, *EIF4H*, *CTBP2*, *PTPRA*, *CNBP*, and *EEF2*) with relatively stable expression levels were identified and selected as new candidate HKGs. Commonly used HKGs including *SDHA* and *YWHAZ* from a previous study, and 2 conventional genes (*ACTB* and *GAPDH*) were also examined. Four different experimental variables: (1) different development stages, (2) hair follicle cycle stages, (3) breeds, and (4) sampling sites were used for determination and validation. Four algorithms (geNorm, NormFinder, BestKeeper, and ΔCt method) and a comprehensive algorithm (ComprFinder, developed in-house) were used to assess the stability of each HKG. It was shown that *NCBP3 + SDHA + PTPRA* were more stably expressed than previously used genes in all conditions analysis, and that this combination was effective at normalizing target gene expression. Moreover, a new algorithm for comprehensive analysis, ComprFinder, was developed and released.

**Conclusion:**

This study presents the first list of candidate HKGs for *C. hircus* skin tissues based on an RNA-seq dataset. We propose that the *NCBP3 + SDHA + PTPRA* combination could be regarded as a triplet set of HKGs in skin molecular biology experiments in *C. hircus* and other closely related species. In addition, we also encourage researchers who perform candidate HKG evaluations and who require comprehensive analysis to adopt our new algorithm, ComprFinder.

## Background

In molecular biology research, determining the relative changes in target gene expression at the transcriptional level requires precise quantitative analysis. The emergence and development of quantitative real-time polymerase chain reaction (qRT-PCR) has enabled comprehensive mRNA quantification. Furthermore, qRT-PCR is a commonly used technique due to its accuracy, sensitivity, reproducibility, and cost-effectiveness in analyzing gene expression [1, 2]. The copy number of nucleic acid was calculated through the changes in real-time fluorescence reaction. The changes is typically reported as a cycle threshold value (Ct) in the comparative Ct method [3]. The qRT-PCR assay relies on house-keeping genes (HKGs) to obtain relative gene expression data [4, 5], thus choosing HKGs has become a major source of error and bottlenecks in qRT-PCR experiments.

In qRT-PCR experiments, inadequate HKG selection may lead to an inappropriate interpretation of target gene expression [6]. There are two common mistakes when selecting HKGs: (I) HKGs are selected based on experience without reviewing HKG research study, and (II) a single HKG with poor stability is used. In recent years, it has been reported with increasing frequency that the commonly used HKGs, such as *ACTB*, *GAPDH*, and *18sRNA,* have critical limitations [7, 8]. Ideal endogenous HKGs should exhibit consistent expression levels across all experimental conditions (e.g. cell types, physiological states, and growth conditions) [9, 10]. Unfortunately, no HKGs are stable across all experimental conditions, which means that each experimental system may need to use unique HKG(s) to accurately explore the specific research question being investigated.

Goat (*Capra hircus*) is an economically important livestock species as a source of meat, hair, and dairy products [11]. Skin tissue, as the largest biological organ with important functions including physical protection from injury and infection, thermal insulation, and providing the substrate for growing hair. To reveal the molecular regulatory mechanism of hair follicle activity, it is necessary to clarify the pattern of target gene expression under different conditions, such as different stages of the hair follicle cycle. Unfortunately, most molecular studies examined goat skin have only included a single HKG such as *ACTB* [12–14] or *GAPDH* [15, 16]. In 2014, Bai et al. [17] selected 10 commonly used HKGs based on a literature review to explore their stability in different hair follicle cycles of Liaoning cashmere goats. However, due to the limited number of animals used and testing only of commonly used HKGs, the previously published study [17] resulted in a limited impact. The development of high-throughput RNA-seq technology provides a method of determining spatiotemporal expression at the transcriptome level, and provides a novel approach for the identification of HKGs [18, 19]. This strategy was successfully used to identify candidate HKGs for *Artemisia sphaerocephala* [7], *Pyropia yezoensis* [20], *Euscaphis* [21], *Arabidopsis pumila* [22], fish [23], tomato leaves [24], and holstein cows [25]. Therefore, we hypothesized that the novel, credible HKGs which serve goat skin research can be predicted and validated via transcriptome sequencing data.

In this study, the transcriptome dataset of 39 goat skin tissue samples was analyzed. Potential HKGs were predicted, of which 8 genes (*SRP68*, *NCBP3*, *RRAGA*, *EIF4H*, *CTBP2*, *PTPRA*, *CNBP*, and *EEF2*) were selected based on their relatively stable expression levels. Four commonly used HKGs (*SDHA*, *YWHAZ*, *ACTB*, and *GAPDH*) were selected for comparison. These 12 genes were amplified using qRT-PCR in four groups with different experimental treatments. Four different algorithms (geNorm [26], ΔCt method [27], NormFinder [28], and BestKeeper [29]) and a comprehensive method (ComprFinder, a newly developed method by our team) were used to evaluate the stability of each HKG. Finally, the reliability of the recommended optimal HKGs was validated and confirmed.

## Results

### Selection of novel candidate HKGs based on RNA-seq data

**Fig. 1:**
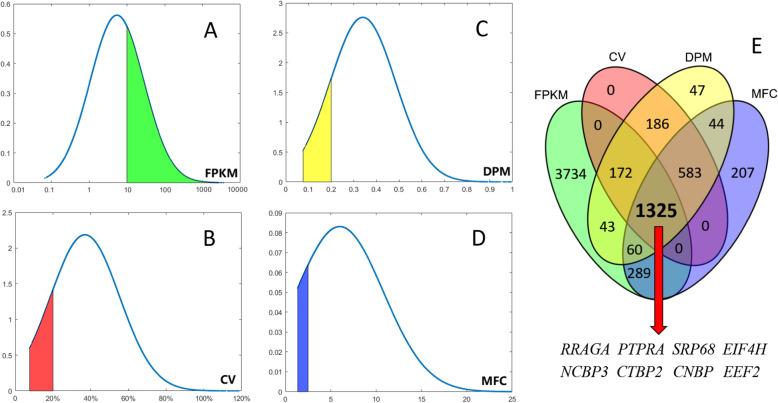
Probability density curve of FPKM, CV, DPM and MFC of 15,853 unigenes. **a**-**d** The y-axes indicate the probability values in all 15,853 genes. **e** The overlap genes were found by the Venn diagram analysis

From a complete transcriptome dataset, the fragments per kilobase of exon model per million mapped reads (FPKM) of all transcripts from each sample were obtained. We first removed some transcripts which did not have a credible function annotation, or exhibited low levels of expression (FPKM = 0). This resulted in 15,853 unigenes being found for further selection. Next, genes with a relatively high expression level (FPKM ≥10 or ≥ the 80th percentile) as determined by the mean FPKM value, and genes with low variability as determined by the coefficient of variation (CV, %), maximum fold change (MFC), and dispersion measure (DPM), were considered (see Methods section). As shown in Fig. 1, the probability density curve of all 15,853 unigenes was evaluated by these 4 indicators.

1. FPKM. Potential HKGs were relatively highly expressed genes [8]. In this study, 5623 genes had FPKM values ≥10 (35.5% of 15,853 genes, the green area in Fig. 1a).
2. CV (%). The most promising HKGs would have the lowest CV values. A total of 2266 genes with a CV ≤ 20% (14.3% of 15,853 genes, the red area in Fig. 1b) were retained in this step with CVs ranging from 7.7 to 20.0%.
3. DPM. Most stable genes exhibited lower DPM values. The default parameter of DPM < 0.3 returned an excessive 7025 unigenes, and so a more stringent DPM < 0.2 was used. Following this, 2026 genes (12.8% of 15,853 genes, the yellow area in Fig. 1c) were retained in this step with DPM values ranging from 0.09 to 0.2.
4. MFC. This parameter reflects the range of extremum value, and the lowest MFC values are preferable. In this study, MFC < 2.5 was used which produced 2508 genes (15.8% of 15,853 genes, the blue area in Fig. 1d), all within the range of 1.35 to 2.5.

A Venn diagram was constructed for the 4-color blocks (green, red, yellow, and blue corresponding to those used in Fig. 1a-d, respectively). This showed that 1325 genes (Fig. 1e) met all 4 of the above requirements, and are significantly enriched in 11 signaling pathways (*q* < 0.05) as shown in Additional file 1: Figure S1. These genes were considered as candidate HKGs and of these, 8 genes (*RRAGA*, *PTPRA*, *SRP68*, *EIF4H*, *NCBP3*, *CTBP2*, *CNBP*, and *EEF2)* that with lower CV value, higher FPKM value, and easier primers design were selected for further qualification. Besides, 4 genes outside of the initial 1325 were considered, including *SDHA* and *YWHAZ* as they had previously been proposed by other researchers [17], and *ACTB* and *GAPDH* genes were included as the most commonly used endogenous HKGs for exploring target gene expression in goats. In total, 12 candidate HKGs were analyzed in subsequent steps. Each gene was ranked based on its CV value with a lower CV receiving a higher-ranking order (Table 1).

**Table 1:** The summarised information of 12 potential HKGs based on transcriptome data

Type	Gene symbol	Mean_FPKM	CV (%)	Ranking order ^a^	MFC ^b^	DPM ^c^
New predicted candidate HKGs	*RRAGA*	51.4	8.4%	6	1.416	0.083
	*PTPRA*	23.8	9.1%	8	1.474	0.090
	*SRP68*	27.2	9.2%	9	1.510	0.091
	*EIF4H*	133.0	9.5%	16	1.479	0.094
	*NCBP3*	10.0	9.5%	17	1.542	0.094
	*CTBP2*	22.5	9.9%	25	1.566	0.098
	*CNBP*	226.5	14.3%	458	1.880	0.141
	*EEF2*	499.7	15.1%	619	1.923	0.149
Suggested by previous study	*SDHA*	44.0	18.5%	1679	2.710	0.182
	*YWHAZ*	137.5	19.2%	1946	2.320	0.189
Conventional HKGs	*ACTB*	556.1	24.6%	4456	2.962	0.239
	*GAPDH*	391.6	29.9%	6855	2.945	0.286

^a^ Ranking order in all genes based on CV value within all 15,853 unigenes

^b^ MFC, maximum fold change, highest/lowest FPKM value of one gene within 39 transcriptome profiles

^c^ DPM, dispersion measure, were determined by PaGeFinder method and an acceptable value should be ≤0.3

### Amplification specificity and efficiency of the candidate HKGs and target genes

A total of 15 primer pairs including 12 candidate HKGs and 3 target genes were designed for qRT-PCR experiments. Detailed information on gene symbol, primer sequence, and amplicon specifications are shown in Additional file 1: Table S1. Amplification efficiency for all 15 genes ranged from 96.4% for *DKK1* to 103.9% for *PTPRA*, and the coefficient of determination (R^2^) varied from 0.9986 to 0.9999. The specificity for each paired primer was validated by the melting curve analysis, which showed a single amplification peak (Additional file 1: Figure S2). Each pair of primers had good specificity and amplification efficiency around 100%.

### Expression profiles of the candidate HKGs

**Fig. 2:**
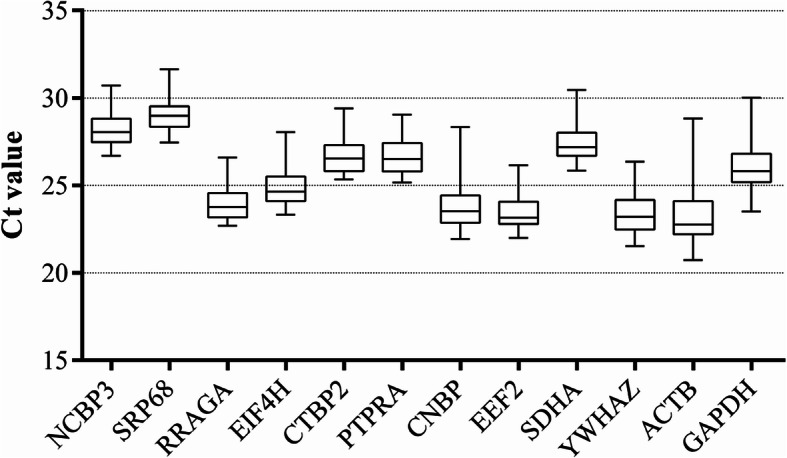
Boxplot of absolute Cq value of the 12 candidate genes in all skin tissue samples. Boxes indicated median (Q2) and quartiles first and third (Q1 and Q3) and whiskers corresponded to the minimum and maximum values

The mean Ct (the average of 3 technical replicates in the same sample) values were used to calculate gene expression levels among samples with distinct experimental factors. As shown in Fig. 2 and Additional file 1: Table S2, the Ct values of the 12 candidate HKGs varied widely from 20.74 to 31.60. The most highly expressed gene was *ACTB* (mean Ct value: 23.25 cycles), and the lowest was *SRP68* (mean Ct value: 29.07 cycles). The top 3 genes with low standard deviations were *SRP68* (0.875), *NCBP3* (0.970), and *PTPRA* (0.972). The 3 most variably expressed genes were *ACTB* (1.483), *CNBP* (1.277), and *GAPDH* (1.258). The narrower standard deviation range of a gene means it has higher expression stability in different samples. Although some genes had a lower standard deviation than others, experimental errors are always possible. Therefore, to obtain a reliable evaluation of these candidate HKGs, further analysis with more scientific algorithms is needed.

### Analysis of HKG expression stability

In this study, 4 publically available algorithms were used to evaluate HKGs for higher-accuracy stability rankings: geNorm, NormFinder, BestKeeper, and the ΔCt method.

#### geNorm analysis

**Fig. 3:**
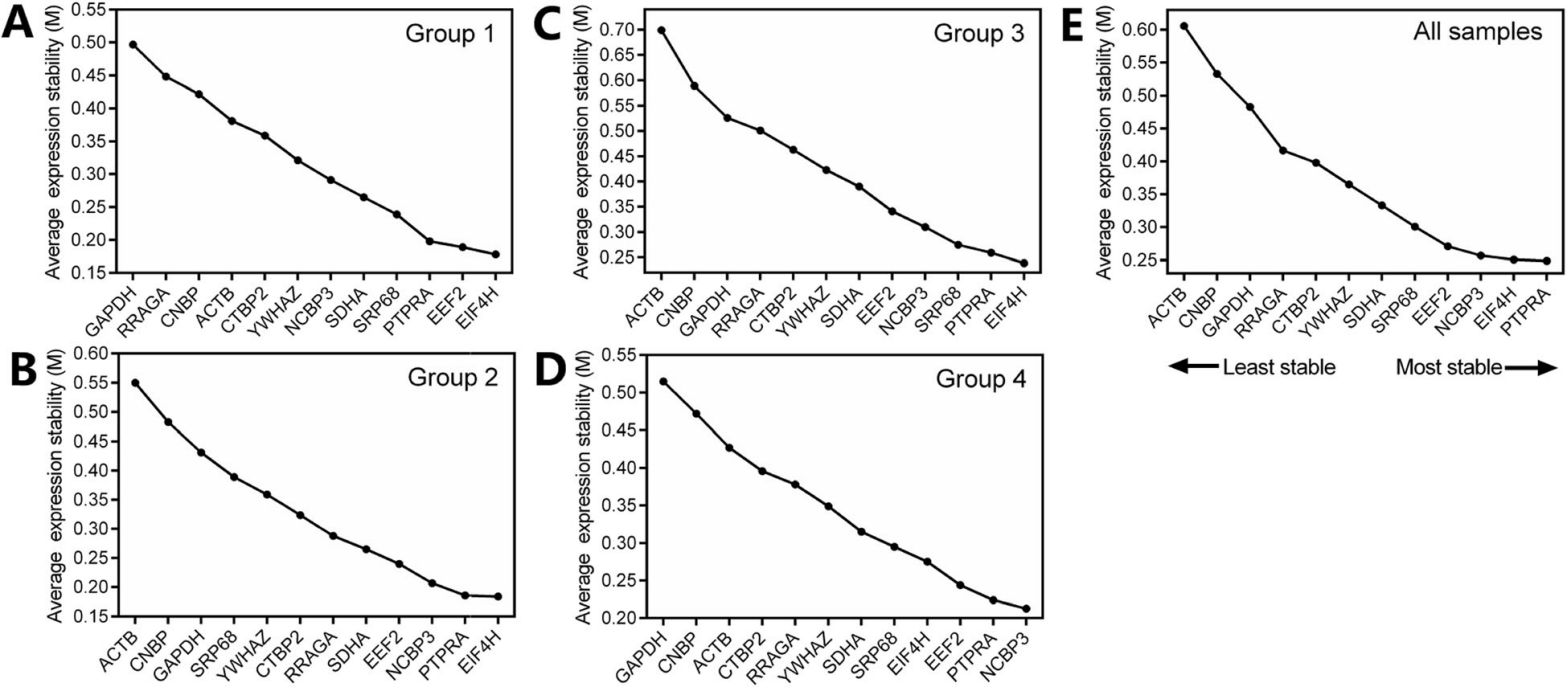
Average expression stability (M-value) calculated by geNorm. **a** Group 1, 4 different development stages; **b** Group 2, 3 time-points in hair follicle cycle; **c** Group 3, 4 goat breeds; **d** Group 4, 5 sampling sites on the body of the goat. **e** All samples including groups 1–4

Gene expression stability was determined by the M-value in geNorm analysis; the lower M value suggests a higher gene expression stability. For group 1, the two most stable genes were *EIF4H* and *EEF2* with the lowest M value, and *GAPDH* was the most unstable gene (Fig. 3a). For group 2, the two most stable genes were *EIF4H* and *PTPRA*, and *ACTB* was the most unstable gene (Fig. 3b). For group 3, the two most stable genes were *EIF4H* and *PTPRA*, whereas *ACTB* was the most unstable gene (Fig. 3c). For group 4, the two most stable genes were *NCBP3* and *PTPRA*, and *GAPDH* was the most unstable gene (Fig. 3d). For all samples, geNorm analysis was conducted on 39 samples and 12 HKGs. It was determined that the 3 most stable genes were *PTPRA*, *EIF4H*, and *NCBP3.* Conversely, *ACTB*, *CNBP*, and *GAPDH* were the most unstable genes (Fig. 3e).

geNorm can be used to determine the minimum optimal number of HKGs needed for accurate normalization under different experimental treatments by analyzing pairwise variation (V_n_/V_n + 1_). This method recognizes V_n_/V_n + 1_ < 0.15 as a threshold value, and “n” as an appropriate number of HKG needed. The V_2_/V_3_ values for all the experimental variables were below the cut-off value of 0.15 (0.067, 0.078, 0.099, 0.091, and 0.081 for group 1, 2, 3, 4, and all samples, respectively), which indicate that using double HKGs (first two genes in each group) is sufficiently accurate for use in normalizing qRT-PCR derived gene expression data (Additional file 1: Figure S3). The triplet or more gene combinations can also be used as V_n_/V_n + 1_ < 0.15 (*n* ≥ 3).

#### NormFinder analysis

Expression stability values, as determined by NormFinder, are shown in Table 2. For group 1, *SDHA* and *EIF4H* were the most stable HKGs, and *ACTB* was the least stable gene, which was the same as was determined by geNorm. In group 2, *SDHA* and *NCBP3* were the most stable HKGs while *ACTB* was the least stable gene. In group 3, *SDHA* and *YWHAZ* got the top rank, while *ACTB* ranked at the lowest. In group 4, *PTPRA* and *NCBP3* were the most stable, while *GAPDH* ranked at the lowest. In all samples, *SDHA* and *NCBP3* were the most stable, while *ACTB* was the least.

**Table 2:** Gene expression stability calculated by NormFinder

Gene name	Group 1	Group 2	Group 3	Group 4	All samples
*SDHA*	0.007(1)	0.006 (1)	0.006 (1)	0.008 (8)	0.009 (1)
*NCBP3*	0.011 (5)	0.007 (2)	0.017 (8)	0.006 (2)	0.011 (2)
*PTPRA*	0.008 (3)	0.011 (5)	0.014 (4)	0.005 (1)	0.012 (3)
*EEF2*	0.012 (7)	0.008 (3)	0.014 (6)	0.006 (3)	0.012 (4)
*CTBP2*	0.013 (8)	0.012 (7)	0.013 (3)	0.007 (6)	0.013 (5)
*EIF4H*	0.008 (2)	0.012 (6)	0.017 (7)	0.007 (4)	0.014 (6)
*YWHAZ*	0.010 (4)	0.017 (10)	0.011 (2)	0.007 (5)	0.015 (7)
*RRAGA*	0.015 (9)	0.010 (4)	0.019 (9)	0.008 (7)	0.015 (8)
*SRP68*	0.011 (6)	0.015 (9)	0.019 (10)	0.009 (9)	0.016 (9)
*GAPDH*	0.019 (11)	0.015 (8)	0.014 (5)	0.014 (12)	0.018 (10)
*CNBP*	0.016 (10)	0.019 (11)	0.026 (11)	0.012 (10)	0.021 (11)
*ACTB*	0.021 (12)	0.021 (12)	0.028 (12)	0.014 (11)	0.026 (12)

#### BestKeeper analysis

The BestKeeper algorithm used std-values to assess HKG stability with the lower the std-value, the more stable HKG expression was. As shown in Table 3, in group 1, *SDHA* and *PTPRA* were the most stable HKGs, whereas *RRAGA* was the least stable. The same was observed with the geNorm analysis. In group 2, *SDHA* and *EEF2* were the most stable HKGs, while *ACTB* was the least stable. In group 3, *SDHA* and *YWHAZ* got the top rank, while *SRP68* ranked at the lowest. In group 4, *EEF2* and *NCBP3* were most stable, while *GAPDH* was the least. In all samples, *SDHA* and *EEF2* were most stable, while *ACTB* was the least.

**Table 3:** Expression stability std-values calculated using BestKeeper

Gene name	Group 1	Group 2	Group 3	Group 4	All samples
*SRP68*	0.468 (1)	0.506 (3)	0.631 (1)	0.767 (3)	0.663 (1)
*SDHA*	0.516 (2)	0.464 (1)	0.737 (3)	0.760 (2)	0.733 (2)
*NCBP3*	0.611 (8)	0.510 (4)	0.743 (4)	0.826 (6)	0.753 (3)
*CTBP2*	0.536 (3)	0.538 (6)	0.776 (6)	0.746 (1)	0.764 (4)
*EIF4H*	0.546 (4)	0.573 (7)	0.709 (2)	0.912 (9)	0.768 (5)
*EEF2*	0.557 (5)	0.473 (2)	0.813 (8)	0.875 (7)	0.775 (6)
*PTPRA*	0.562 (6)	0.615 (9)	0.758 (5)	0.822 (5)	0.779 (7)
*RRAGA*	0.693 (9)	0.530 (5)	0.789 (7)	0.820 (4)	0.811 (8)
*YWHAZ*	0.586 (7)	0.742 (12)	0.856 (10)	0.901 (8)	0.871 (9)
*CNBP*	0.778 (11)	0.583 (8)	0.987 (11)	0.986 (12)	0.962 (10)
*GAPDH*	0.948 (12)	0.674 (10)	0.837 (9)	0.981 (11)	0.984 (11)
*ACTB*	0.719 (10)	0.679 (11)	1.203 (12)	0.978 (10)	1.114 (12)

#### ΔCt analysis

The 12 candidate HKGs were analyzed using the Delta Ct method, the data of which is presented in Table 4. The stability of the gene is inversely related to the std-value, thus a lower value indicates greater stability. In group 1, the two most stably expressed genes were *PTPRA* and *SDHA*, and the lowest were *GAPDH* and *ACTB*. In group 2, the two most stable genes were *EEF2* and *SDHA*, and the least were *ACTB* and *CNBP*. In group 3, *SDHA* and *PTPRA* were the most stably expressed, whereas *ACTB* and *CNBP* were the least. In group 4, the top two stably expressed genes were *NCBP3* and *EEF2*, whereas *CNBP* and *GAPDH* were the least. In all samples, the 3 most stable genes were *PTPRA*, *EEF2*, and *SDHA*, while *GAPDH*, *CNBP*, and *ACTB* were the least stable genes.

**Table 4:** Gene expression stability calculated by the ΔCt method

Gene name	Group 1	Group 2	Group 3	Group 4	All samples
*PTPRA*	0.391 (1)	0.439 (4)	0.573 (2)	0.443 (3)	0.499 (1)
*EEF2*	0.423 (4)	0.412 (1)	0.587 (4)	0.428 (2)	0.500 (2)
*SDHA*	0.391 (2)	0.417 (2)	0.535 (1)	0.475 (4)	0.503 (3)
*NCBP3*	0.457 (6)	0.424 (3)	0.643 (7)	0.422 (1)	0.512 (4)
*EIF4H*	0.392 (3)	0.441 (5)	0.604 (5)	0.483 (6)	0.520 (5)
*CTBP2*	0.486 (8)	0.516 (7)	0.619 (6)	0.493 (8)	0.553 (6)
*YWHAZ*	0.425 (5)	0.549 (8)	0.583 (3)	0.486 (7)	0.557 (7)
*RRAGA*	0.573 (10)	0.442 (6)	0.672 (8)	0.479 (5)	0.568 (8)
*SRP68*	0.460 (7)	0.583 (9)	0.702 (10)	0.499 (9)	0.590 (9)
*GAPDH*	0.740 (12)	0.594 (10)	0.699 (9)	0.731 (12)	0.741 (10)
*CNBP*	0.555 (9)	0.690 (11)	0.957 (11)	0.644 (11)	0.757 (11)
*ACTB*	0.582 (11)	0.826 (12)	1.198 (12)	0.597 (10)	0.973 (12)

#### A comprehensive ranking of the four methods examined

Next, the ComprFinder algorithm was employed to obtain a comprehensive score that was used to rank the potential HKGs (Table 5). In group 1, the 3 most stable HKGs were *EIF4H*, *PTPRA*, and *SDHA*. In group 2, *SDHA*, *NCBP3*, and *EEF2* were the most stable HKGs analyzed. In group 3, *SDHA*, *PTPRA*, and *EIF4H* were the three most stable HKGs analyzed. In group 4, *NCBP3*, *PTPRA*, and *EEF2* were the most stable genes. The overall rankings, from the highest to the lowest stability, were *NCBP3* > *SDHA* > *PTPRA* > *EEF2* > *EIF4H* > *SRP68* > *CTBP2* > *YWHAZ* > *RRAGA* > *GAPDH* > *CNBP* > *ACTB*. It is interesting to note that the top 3 genes in different group rankings have at least 2 of *NCBP3*, *SDHA*, and *PTPRA*. In contrast, the commonly used HKGs, *ACTB*, and *GAPDH,* were relegated to the bottom 2 and 4 positions, respectively.

**Table 5:** Comprehensive rankings calculated using the ComprFinder method

Ranking No	Group 1		Group 2		Group 3		Group 4		All samples	
	Gene	Score	Gene	Score	Gene	Score	Gene	Score	Gene	Score
1	*EIF4H*	0.063	*SDHA*	0.059	*SDHA*	0.129	*NCBP3*	0.105	*NCBP3*	0.096
2	*PTPRA*	0.090	*NCBP3*	0.082	*PTPRA*	0.170	*PTPRA*	0.105	*SDHA*	0.099
3	*SDHA*	0.093	*EEF2*	0.090	*EIF4H*	0.180	*EEF2*	0.193	*PTPRA*	0.108
4	*EEF2*	0.171	*EIF4H*	0.210	*SRP68*	0.230	*SDHA*	0.211	*EEF2*	0.129
5	*SRP68*	0.174	*RRAGA*	0.227	*EEF2*	0.247	*SRP68*	0.245	*EIF4H*	0.143
6	*YWHAZ*	0.256	*PTPRA*	0.236	*NCBP3*	0.252	*CTBP2*	0.263	*SRP68*	0.192
7	*NCBP3*	0.282	*CTBP2*	0.322	*YWHAZ*	0.277	*EIF4H*	0.309	*CTBP2*	0.248
8	*CTBP2*	0.358	*SRP68*	0.430	*CTBP2*	0.293	*RRAGA*	0.327	*YWHAZ*	0.311
9	*RRAGA*	0.605	*GAPDH*	0.609	*GAPDH*	0.399	*YWHAZ*	0.361	*RRAGA*	0.320
10	*CNBP*	0.637	*YWHAZ*	0.630	*RRAGA*	0.404	*ACTB*	0.795	*GAPDH*	0.603
11	*ACTB*	0.677	*CNBP*	0.697	*CNBP*	0.730	*CNBP*	0.820	*CNBP*	0.680
12	*GAPDH*	0.971	*ACTB*	0.943	*ACTB*	1.000	*GAPDH*	0.994	*ACTB*	1.000

*NCBP3*, *SDHA*, *PTPRA* were the 3 most stable HKGs across all samples with scores within a tight range, calculated final score (FS) of 0.096, 0.099, and 0.108, respectively. They were also preferably ranked in groups 1–4 relative to other genes and were therefore considered to be the 3 most promising candidate HKGs, and were advanced for further validation.

### Validation of the recommended HKGs by *DKK1*, *SHH*, and *FGF5* genes

Based on the above analyses, 3 target genes (*DKK1*, *SHH*, and *FGF5*) were further characterized based on their changes in expression levels during the secondary hair follicle cycle (T1, T2, T3) with normalizations using different single HKG and multi-gene combinations. It was observed that *NCBP3*, *SDHA*, and *EEF2* were the top 3 HKGs in group 2 (factor: hair follicle cycle) based on their ComprFinder FS values. Therefore, it can be concluded that the combination of *NCBP3 + SDHA + EEF2* was the best-normalized gene set for group 2. Since these 3 genes (*NCBP3*, *SDHA*, and *PTPRA*) are possibly the most important candidate HKGs, they were further characterized to determine optimal combinations for normalization of gene expression studies. Four multi-gene combinations, including *NCBP3 + SDHA + PTPRA*, *NCBP3 + SDHA*, *NCBP3 + PTPRA*, and *SDHA + PTPRA,* in addition to 3 single-genes (*NCBP3*, *SDHA*, and *PTPRA)* were added to this analysis. Conversely, *ACTB* and *GAPDH* were used for comparison and were also examined as the multi-gene combination *ACTB* + *GAPDH*. In total, 11 multi-gene combinations or single genes were used as normalization factors.

**Fig. 4:**
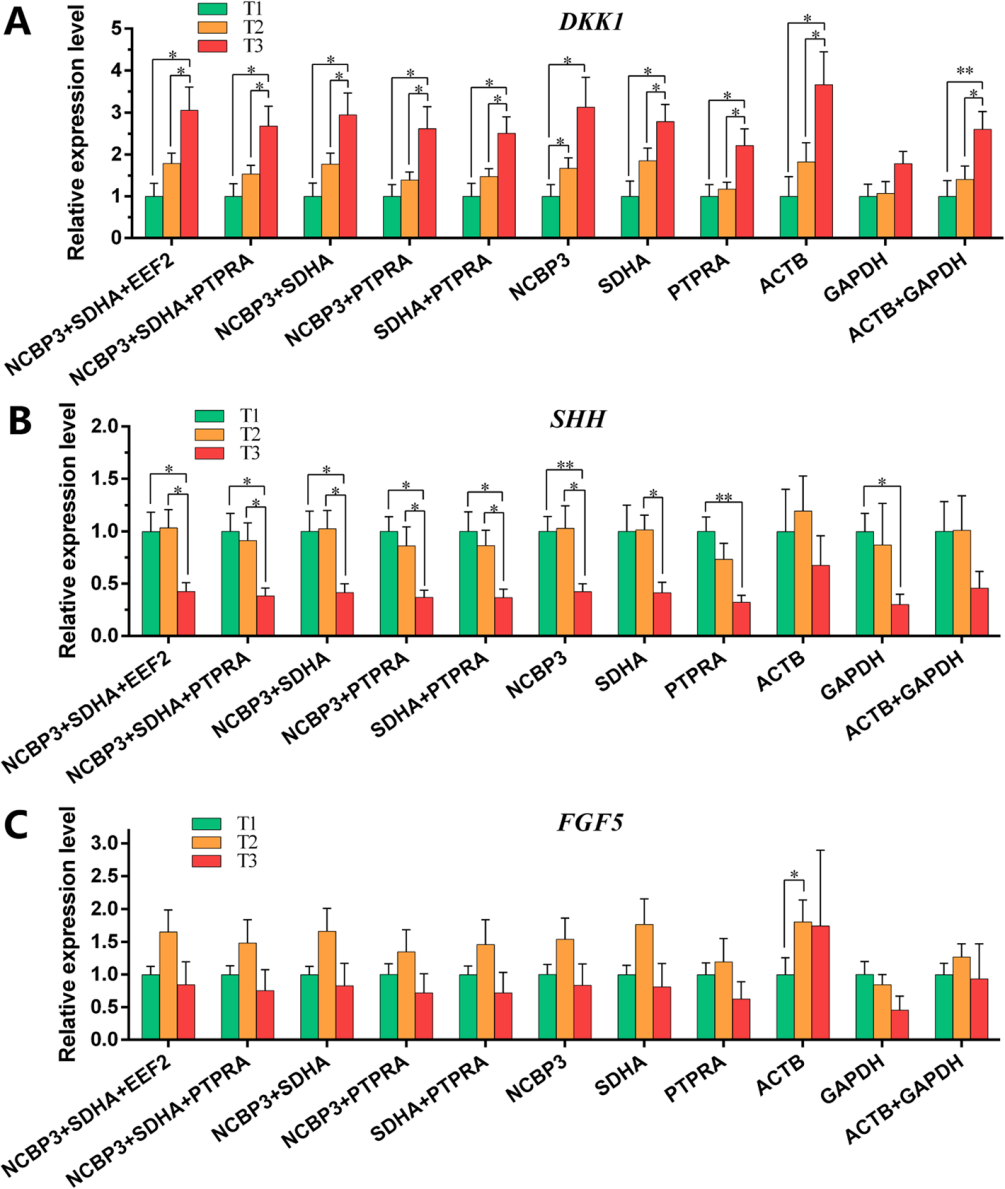
Relative gene expression levels normalized by 11 types of single or multiple gene combinations of HKGs. Expression of *DKK1* (**a**), *SHH* (**b**), and *FGF5* (**c**) were normalized by the most stable single or multiple gene combinations (*NCBP3* + *SDHA* + *EEF2*, *NCBP3* + *SDHA* + *PTPRA*, *NCBP3* + *SDHA*, *NCBP3* + *PTPRA*, *SDHA* + *PTPRA*, *NCBP3*, *SDHA*, *PTPRA*,) and the most unstable single or multiple genes combination (*ACTB*, *GAPDH*, *ACTB* + *GAPDH*). The error bars represent the SEM, and the paired t-test in any two stages, (*P < 0.05, **P < 0.01, *n* = 6) for each hair follicle cycle time-point of IMCG. T1, T2, and T3 indicate the anagen, catagen, and telogen, respectively

As is shown in Fig. 4a, the expression profiles of *DKK1* were similarly obtained using the 8 stable single-gene and multi-gene combinations. Furthermore, it was observed that *DKK1* was more highly expressed in T2 compared to T1, and it was most highly expressed during the T3. Among the unstable single- and multi-gene combinations, only *ACTB* and *ACTB + GAPDH* performed similarly to the stable genes. However, the gene expression profile as normalized by *GAPDH* was different from the other conditions, and no significant difference has been identified among T1, T2, and T3. Expression of the *SHH* gene was even during the T1 and T2, but there was a significant decrease in T3 (Fig. 4b). The 5 multi-gene combinations and *NCBP3*, *SDHA* identified this trend, but *PTPRA* did not. Though the *GAPDH*-normalized gene expression profile had similar trends to stable multi-gene combinations, *ACTB* was different. The combination of *ACTB + GAPDH* identified this expression change as a trend, but was not able to detect significant changes in expression. The expression profile of the *FGF5* gene, when normalized by the most stable candidate HKGs used individually or in combination here, were very similar. High expression levels were observed in T2, but no statistical significance was identified relative to T1 and T3 (Fig. 4c). The combination of *ACTB + GAPDH* showed a similar pattern to the stable HKGs, but when *ACTB* and *GAPDH* were used individually, the expression patterns were completely different. Furthermore, significant differences in *ACTB* were also identified in T2 relative to T1.

**Fig. 5:**
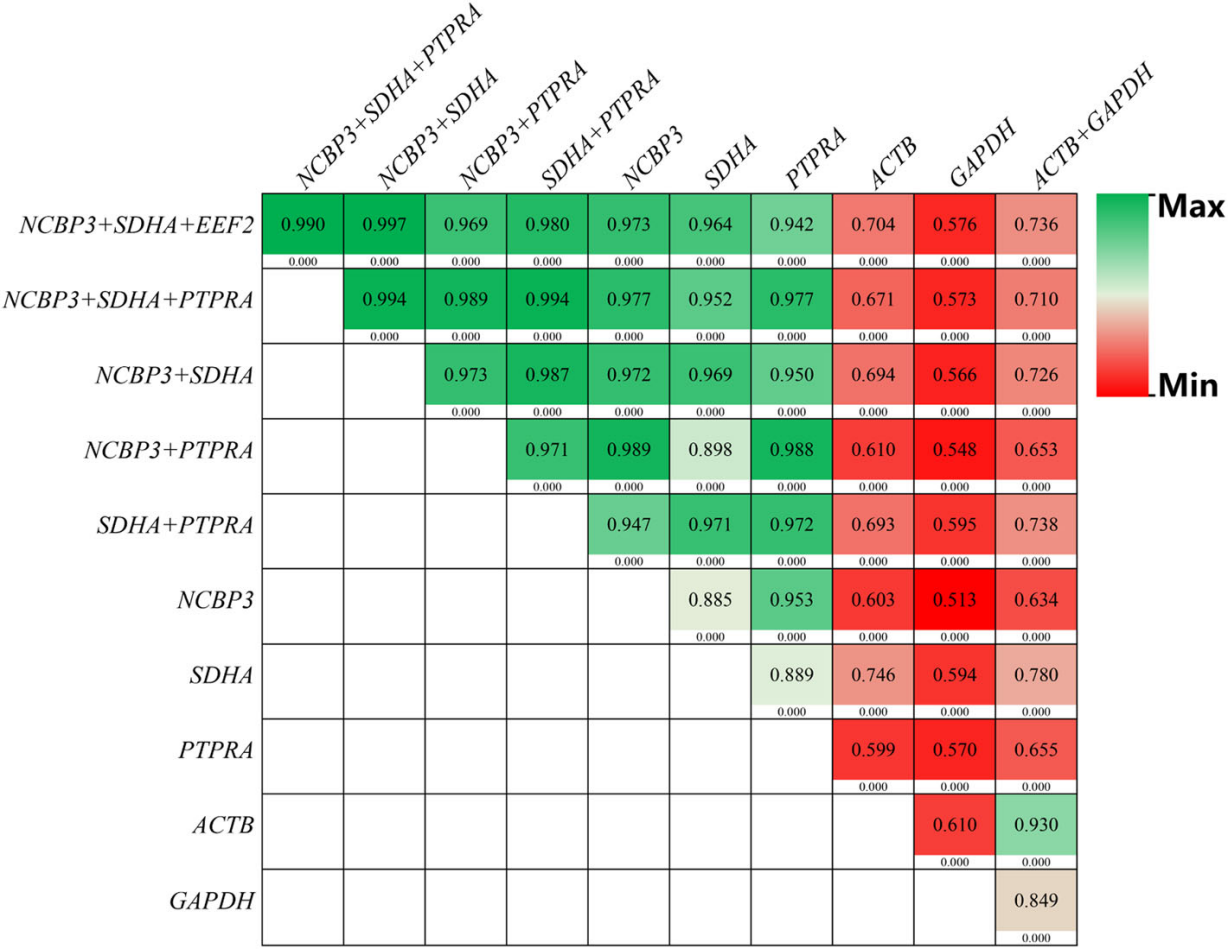
Heat map of correlation coefficients of relative gene expression levels based on different normalized HKGs. Three target genes were detected in 18 skin samples and normalized by different types of HKGs. The number in each color block is the correlation coefficient (R-value), and the number below the color block is the *P*-value of the corresponding R-value. The sample size was 54 (3 target genes*18 skin samples) for each color block

The above-mentioned results derived from Fig. 4 reflect the differences of expression profiles of a single target gene normalized by 11 types of single or multiple-gene combinations. To further understand the relationship of those single or multi-HKG combinations, a correlation analysis on these relative expression data (2^-ΔCt^) of 3 target genes was performed. As shown in Fig. 5, the normalized results using *NCBP3 + SDHA + EEF2* and *NCBP3 + SDHA + PTPRA* had a high correlation coefficient (*R* = 0.990, *P* < 0.001), suggesting that they have extremely similar normalization capabilities. Other double-gene combinations including *NCBP3 + SDHA*, *NCBP3 + PTPRA*, and *SDHA + PTPRA* had high correlation coefficients, ranging from 0.969–0.997 with *NCBP3 + SDHA + EEF2*. Also, these double-gene combinations had high correlation coefficients of 0.989–0.994 with *NCBP3 + SDHA + PTPRA*. This indicated that these 3 types of double-gene combinations exhibited similar normalization capabilities to *NCBP3 + SDHA + EEF2* and *NCBP3 + SDHA + PTPRA*. For single stable HKGs, *NCBP3*, *SDHA*, and *PTPRA* also exhibited high correlation coefficients with *NCBP3 + SDHA + EEF2* (0.942–0.973) and *NCBP3 + SDHA + PTPRA* (0.952–0.977). The *ACTB*, *GAPDH*, and *ACTB + GAPDH* combinations had relatively low correlation coefficients with any of the stable single- (0.513–0.780) and multi-gene combinations (0.548–0.738).

## Discussion

### Standard criteria for HKG screening for skin tissue research in goats

#### Which candidate HKGs should we choose?

Four original algorithms were used to identify the expression stability values of 12 candidate HKGs and their FS values were determined using a comprehensive algorithm. However, even for the final ComprFinder value, the results varied between different groups. If the top 3 genes were considered, groups 1–4 should theoretically be *EIF4H + PTPRA + SDHA*, *SDHA + NCBP3 + EEF2*, *SDHA + PTPRA + EIF4H*, and *NCBP3 + PTPRA + EEF2*, and a total of 5 HKGs (*EIF4H*, *PTPRA*, *SDHA*, *NCBP3*, *EEF2*) would be needed in goat skin research. In theory, it is preferable to use multiple high-performing HKGs as a normalization factor. However, in practice, the additional cost and excessive number of HKGs, limit the number of samples that can be tested. Therefore, the minimum number of HKGs should be used to meet the relevant statistical needs, in addition to reducing experimental costs [10, 30]. In this study, *NCBP3*, *SDHA*, and *PTPRA* were the top 3 most stable HKGs for all samples, and 2 of those ranked in the top 3 of each group. They were therefore considered as the most three stably expressed HKGs, and for further validation.

#### How many candidate HKGs should be used?

There is still no specific theory prescribing a certain number of HKGs to be used. In the above discussion, *NCBP3*, *SDHA*, and *PTPRA* were proposed for their excellent stability, however, which single or multiple gene combinations (*NCBP3* + *SDHA* + *PTPRA*, *NCBP3* + *SDHA*, *NCBP3* + *PTPRA*, *SDHA* + *PTPRA*, *NCBP3*, *SDHA*, *PTPRA*) should be used? Compared with the performance of *NCBP3* + *SDHA* + *EEF2* or *NCBP3* + *SDHA* + *PTPRA*, the detection efficacy of *NCBP3* (as shown in Fig. 4a), *SDHA* (as shown in Fig. 4b), and *PTPRA* (as shown in Fig. 4b-c) were not consistent. Considering that the single gene performances were not good, it is recommended that single HKGs should be avoided, even if they were the top-ranked HKGs.

For the double gene combinations *NCBP3* + *SDHA*, *NCBP3* + *PTPRA*, and *SDHA* + *PTPRA* (as shown in Fig. 4a-c), similar expression patterns and detection efficacy were observed as the *NCBP3* + *SDHA* + *EEF2* or *NCBP3* + *SDHA* + *PTPRA* combinations. It was shown that compared to *NCBP3* + *PTPRA* and *SDHA* + *PTPRA*, *NCBP3* + *SDHA* yielded similar results as the *NCBP3* + *SDHA* + *EEF2* or *NCBP3* + *SDHA* + *PTPRA* combinations, possibly because *NCBP3* and *SDHA* were among the top in the final stability ranking in group 2. This also implies that *SDHA + PTPRA*, *SDHA + PTPRA*, and *NCBP3 + PTPRA* may be the optimal double gene combinations for groups 1, 3, and 4, respectively. There are still 3 genes, *NCBP3*, *SDHA*, and *PTPRA*, and there is no type of double gene combination able to cope with multiple factors (groups 1–4).

Considering that the 3-gene combination of *NCBP3 + SDHA + PTPRA* exhibited better stability, it can be applied to various factors in goat dermatologic research, and 3 HKGs is still an acceptable number for qRT-PCR experiments. Therefore, it is recommended that *NCBP3 + SDHA + PTPRA* be adopted as the HKG combination for skin research in goats.

#### The HKGs of skin tissue in goats and other species

As noted previously, common HKGs used in traditional skin research of goats were either *ACTB* [12–14] or *GAPDH* [15, 16], so erroneous data might be obtained. Therefore, the advantage of a set of appropriate HKGs is very valuable. Regarding target genes that have undergone significant changes, these can be identified by less stable HKGs. But for target genes that show slight changes, these can only be identified by optimal HKGs [30]. As far as we know, only one previous HKG study reported on goat skin tissue, Bai et al. [17] selected ten commonly used HKGs by consulting the literature. The selected HKGs were tested on 3 stages of hair follicle cycle in Liaoning cashmere goats (referred to here as T1, T2, and T3 of IMCG), and authors finally recommended the *SDHA + YWHAZ + UBC* as the HKGs. But their geNorm values (V_2_/V_3_ = 0.159 and V_3_/V_4_ = 0.144) imply that the combination of 3 genes was not ideal. In the present study, large number of biological samples were provided for determination and validation, and multiple algorithms were used for evaluation, with the RNA-seq dataset was used for prediction and selection. Therefore, in terms of both the number and quality of HKGs, this study is a significant stepforward from previous studies.

When studying the target gene expression level in skin tissue from other species, such as Angora rabbits [31], mink [32], mice [33], and humans [34], *ACTB* or *GAPDH* are generally used as the HKGs. The selection of HKGs from the same type of tissue within neighboring species has been widely recognized and accepted. Thus, the data presented here could prove HKGs are suited to skin tissue research, not only for goats, but also for other species.

### Selection and validation of HKGs based on RNA-seq data

Selection and validation of HKGs using RNA-seq produced more reproducible results, had greater sensitivity, yielded better correlation with protein expression levels, and had more accurate detection and higher coverage [35]. To the best of our knowledge, this study is the first one to report on the selection and validation of novel HKGs for qRT-PCR analysis in goats. Two novel HKGs (*NCBP3* and *PTPRA*) and a known HKG (*SDHA*) belonging to the *NCBP3 + SDHA + PTPRA* combination were recommended. Using a similar approach as other studies [19–21], new and improved HKGs were identified through analyzing an RNA-seq dataset. While this study demonstrated the advantages of using RNA-seq datasets in the discovery of new HKGs, it is also possible that the prediction of HKGs by RNA-seq datasets may be lacking in some respects. For example, the ranking order of these candidate HKGs (Table 1) and the determined FS (Table 5) did not match (compared in Additional file 1: Table S3). Specifically, the CV values of *RRAGA* and *SRP68* were in the top 3, although in the final ranking they did not appear in the top 3 positions of any group (groups 1–4 and all samples). This might be the reason why RNA-seq samples in the selection stage and the qRT-PCR samples used in the determination stage did not completely overlap. This phenomenon is consistent with those reported by Gao et al. [20] and implies that the HKGs predicted by RNA-seq screening were not fully reliable, and need further validation by qRT-PCR experiments.

As mentioned before, *ACTB* and *GAPDH* are currently the most popular HKG in the literature, but their limited normalizing capacity was verified here. This suggests that scientists must be cautious when selecting traditional HKGs, especially when identifying target genes that have slight changes in expression. Therefore, it is recommended that common HKGs be included as a comparison, to provide direct evidence. Of course, it must also be acknowledged that mining reliable HKGs require scientific experimental design, complete experimental materials, more algorithmic tools, and a certain amount of scientific research time [30], but these are not available to every research laboratory. Therefore, those experimental systems that do not meet the above conditions were recommended to search for HKGs in close species using the ICG platform [36]. Meantime, use as many HKGs as possible and calculate their arithmetic mean as the normalization factor to increase experimental stability, instead of simply using a single HKG such as *ACTB* or *GAPDH*.

### Comprehensive analysis

#### The requirement of comprehensive analysis, and the shortcomings of the previous algorithms

After evaluating candidate HKGs with the above-mentioned algorithms (geNorm [26], NormFinder [28], BestKeeper [29], and the ΔCt method [27]), it is not surprising that the rankings of candidate genes may vary depending on the algorithm used [30]. Thus, another algorithm is needed for comprehensive ranking. After reviewing the literature on HKGs, it was determined that 2 types of comprehensive algorithms were mainly used: (1) the primary ranking order was used to calculate the arithmetic average, and then get the final ranking [24, 37]; (2) using the primary ranking order, the geometric average is then calculated to get the final ranking. RefFinder [38] is a typical representative (https://www.heartcure.com.au/reffinder/?type=reference) and many studies [7, 19, 20, 22, 39–41] have used it (Times Cited: 352, on Web of Science, 2020/2/3), which illustrates its vast impact. Both the above comprehensive algorithm types depend on the ranking number of the original algorithms. Due to this, it is possible that the use of ranking numbers may cause some errors.

**Fig. 6:**
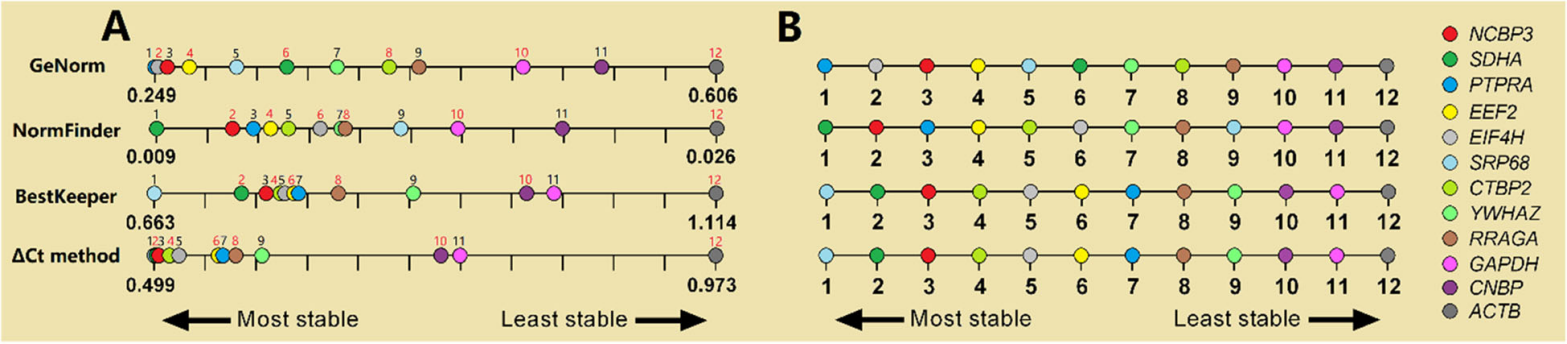
Gene stability values and rank order. The same color presents the same gene. **a** Twelve candidate HKGs unevenly distributed on the axis, this is their true distribution; **b** Twelve candidate HKGs evenly distributed on the axis ordered by 1–12

These ranking numbers reflect the true size of stability values of these candidate HKGs, but they should not be used as the input numerical value for the next calculation. Doing so would excessively reduce or enlarge the real differences among them. Referring to our experimental data as an example, these candidate HKGs were evaluated and unevenly distributed (Fig. 6a) on the axis of the four algorithms. The RefFinder algorithm provides them with the uniform rank of 1–12 (Fig. 6b), and then calculates the geometric average for each candidate HKG. The RefFinder algorithm increases the gap between *PTPRA* and *EIF4H,* and reduces the gap between *CNBP* and *ACTB* (the axis of the geNorm algorithm in Fig. 6a and b). We consider this was unreasonable for the comprehensive evaluation and therefore, in this study, a new algorithm for comprehensive analysis was developed.

#### The ComprFinder algorithm

ComprFinder was intentionally developed to replace RefFinder and the comparison of the two algorithm’s results are shown in Additional file 1: Table S4. It can be seen that the FS and ranking order of the 12 candidate HKGs calculated using the two algorithms are different. For example, *NCBP3* (0.096), *SDHA* (0.099), and *PTPRA* (0.108) were the top 3 genes for all samples calculated by ComprFinder, whereas *PTPRA* (2.14), *SDHA* (2.45), and *EEF2* (3.13) were the top three calculated by RefFinder. The ComprFinder algorithm directly standardizes the results of the original algorithms, unlike RefFinder, which uses the ranking numbers of original algorithms. Theoretically, if the series of HKG expression stability values calculated by each original algorithm is uniformly distributed (likely does not exist), the results of ComprFinder and RefFinder algorithms will be the same. Using the ranking numbers, the real differences among the candidate HKGs will excessively reduce or increase. Therefore, the new comprehensive algorithm can overcome the intrinsic errors caused by artificial assignment. Standardized processing allows the results of different algorithms to have the same dimension and makes them essentially comparable. Finally, the standardized results can be integrated to get a series of scores and a final evaluation. Therefore, ComprFinder would be a more reasonable algorithm than RefFinder for the comprehensive evaluation of HKGs.

We provide a ComprFinder algorithm tool (Additional file 2) for researchers who have comprehensive evaluation needs for candidate HKGs. This tool is based in Microsoft Excel and can be downloaded from the supplementary materials of this article. Briefly, after inputting the original algorithm results into the input area, the ComprFinder algorithm automatically processes the data and all candidate HKGs will be scored and presented in the output area. Although the use of ComprFinder in this study was based on geNorm, BestKeeper, NormFinder, and the ΔCt method, analysis is not limited to these 4 algorithms.

## Conclusion

In this study, we present the first list of candidate HKG selection for goat skin tissue based on transcriptome data. The *NCBP3 + SDHA + PTPRA* combination was identified and recommended as the triplet HKGs for skin molecular biology studies in goats and other closely related species. In addition, a comprehensive algorithm tool based in Microsoft Excel, ComprFinder, was developed for the comprehensive evaluation of candidate HKGs.

## Methods

### Animals and skin tissue samples

All animals and sampling procedures in this study were supervised and approved by the Institutional Animal Care and Use Committee of Southwest University. Each 1 cm^2^ skin tissue was sampled from the Inner Mongolia cashmere goat (IMCG), Dazu black goat (DBG), Hechuan white goat (HCWG), or the first filial generation (F_1_, DBG♂ × IMCG♀). Detailed information regarding the animal source, method of the animal sacrificed, anesthesia procedure, sampling procedure, and sample preservation is found in Additional file 1: Tables S5 and S6. All samples were stored at − 80 °C until further usage.

**Fig. 7:**
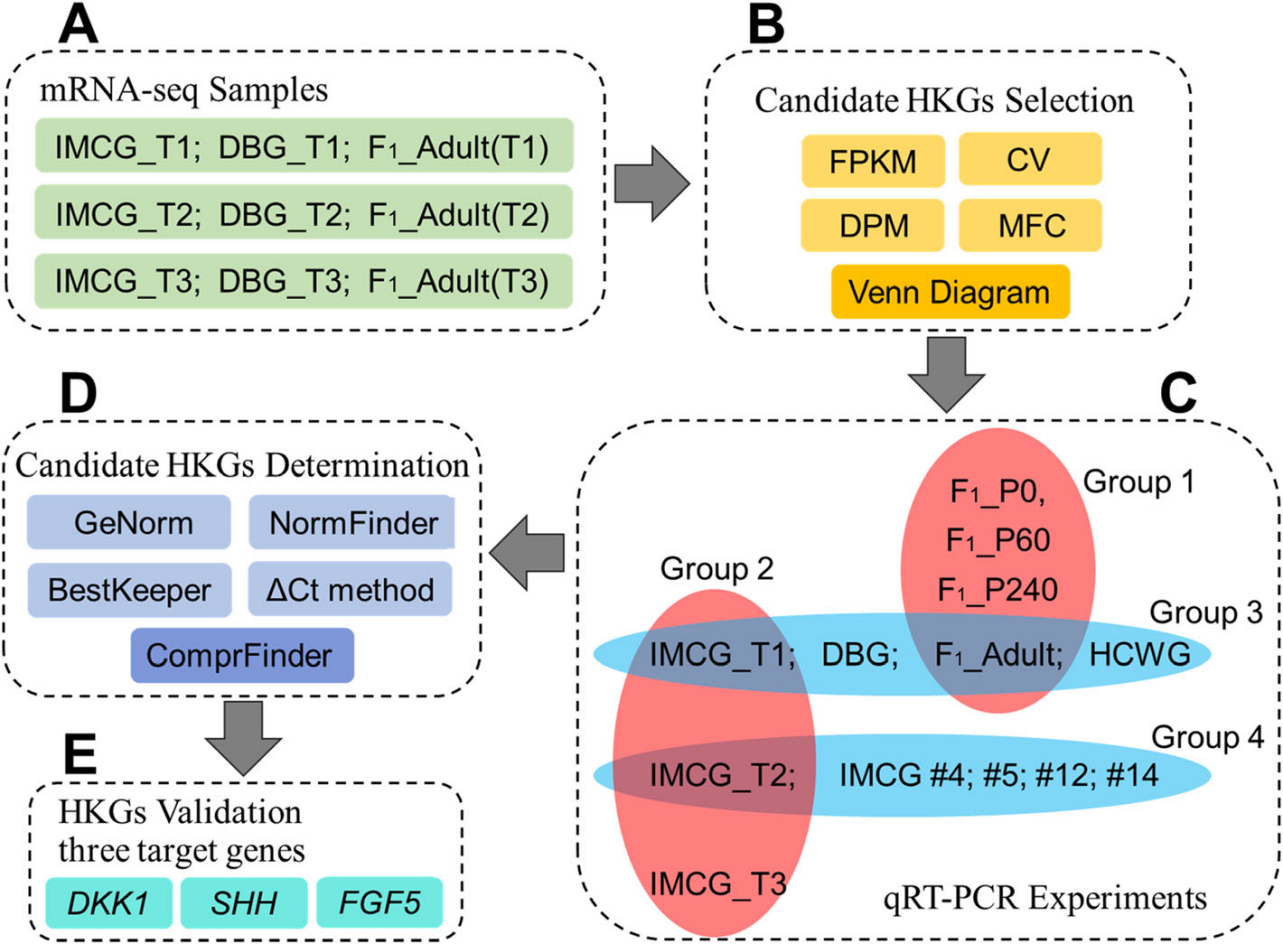
The workflow of this study. **a** The sample information of transcriptome sequence data of these IMCG (*n* = 3*3), DBG (*n* = 3*3) and F1_Adult (*n* = 7*3); **b** Candidate housekeeping genes were preliminarily selected by four indicators which including FPKM, CV, DPM, and MFC, and were further selected by Venn diagram analysis; **c** The sample information of the qRT-PCR experiments on the 4 experimental groups, with 3 biological replicates in every level of each group. Group 1, different development stages (*n* = 4*3); Group 2, hair follicle cycle stages (*n* = 3*3); Group 3, breeds (*n* = 3*3); Group 4, sampling sites (*n* = 5*3). **d** Candidate housekeeping genes were determined using 4 algorithms, including geNorm, NormFinder, BestKeeper and the ΔCt method. An additional comprehensive analysis was conducted using ComprFinder, a new algorithm developed by the authors. **e** The selected HKGs were validated by 3 target genes, and were performed on 18 skin samples (*n* = 6*3)

A total of 48 skin tissue samples were collected to determine (determination stage) and to validate (validation stage) the potential HKGs. The sample sizes (n) for every group (factor) and every level were described in the legend of Fig. 7. In the determination stage of this study (Fig. 7c, Additional file 1: Table S5), all samples were collected from does. Four groups were used, including age (4 development stages, group 1), sampling time (3-stages of hair follicle cycle, group 2), breed (4 different breeds, group 3), and sampling site (5 different sampling sites on the body of the goat, group 4). As shown in Fig. 7c, group 1 included F_1__P0, F_1__P60, F_1__P240, and F_1__Adult, which were sampled at 0-day, 2 months, 8 months, and 2 years after birth from F_1_. Group 2 included IMCG_T1, IMCG_T2, and IMCG_T3, which were sampled during the anagen (September), catagen (December), and telogen (March) from IMCG. Group 3 consisted of animals from 4 breeds and included IMCG, DBG, F_1_, and HCWG. Skin samples from each of the breeds were sampled in the anagen phase of the hair follicle cycle. Skin biopsies collected from group 4 (#4, #5, #6, #12, and #14 from IMCG, as described in our previous publication [42]), were taken from the forearm, dorsal chest, lateral chest, thigh, and the inner side of the forearm as described in our previous study. Except for samples #4, #5, #12, and #14, all samples analyzed here were collected from the lateral chest of the goat body. Except for samples belonging to F_1__P0, F_1__P60, and F_1__P240, all samples were collected from adult goats. In the validation stage of this study (Fig. 7e, Additional file 1: Table S6), 3 bucks of IMCG were added to group 2, to enhance validation accuracy.

### RNA isolation and cDNA synthesis

Total RNA was extracted using the RNAiso Plus kit (#9109, TaKaRa, China) according to the manufacturer’s instructions. The concentration and purity were determined using the Nanodrop2000 (Thermo, USA) with the 260/280 ratios being between 1.8 and 2.0, and the 260/230 ratios were greater than 1.6 in all analyzed RNA samples. First-strand cDNA was synthesized using the 5X All-In-One RT MasterMix (with AccuRT Genomic DNA Removal Kit) (#G492, ABM, Canada) and 1:4 volumes of DEPC water was added to dilute the samples.

### Selection of candidate HKGs

Transcriptome sequencing data of 39 goat skin tissues (unpublished data, Fig. 7a) was performed using the paired-end sequencing technology on an Illumina NovaSeq 6000 platform. After assembly and annotation, the gene expression profiles and read counts of unique transcripts were converted into FPKM values on the platform BMKCloud (www.biocloud.net), according to the formula FPKM = cDNA fragments/[mapped fragments (millions) × transcript length (kb)]. Based on the FPKM value of every gene in each transcriptome, the CV and MFC were calculated using Microsoft Excel and DPM was calculated using a jar package [43]. The CV is defined as the ratio of the SD_FPKM_ to the mean of the FPKM of all samples for one gene. The DPM parameter was introduced for the identification of the HKGs on pattern gene finder (PaGeFinder) [43], and their jar package was downloaded and run from the PaGeFinder website. The MFC, which is defined as the fold change between the largest and smallest FPKM values within 39 RNA-seq were calculated. The standard criteria for HKGs are relatively high expression level, and low expression variation, therefore, a candidate HKG should have a relatively high FPKM value, and low CV, DPM and MFC values.

Genes with RPKM, CV, MFC, and DPM fulfilling the criteria of HKGs were retained for further analysis (Fig. 7b). Moreover, two HKGs (*SDHA* and *YWHAZ*) from a previous study by Bai [17], and two commonly used HKGs (*ACTB* and *GAPDH*) were also considered as HKGs. All candidate HKGs were amplified using qRT-PCR for subsequent determination and validation. The probability density curve was drawn by an in house script (Additional file 3) using the Matlab software (https://ww2.mathworks.cn/products/matlab.html). Venn diagram analysis was performed using the OmicShare online platform tools (http://www.omicshare.com/tools).

### Primer design and amplification efficiency analysis

Specific primers were designed using the Primer-BLAST [44] web tool (https://www.ncbi.nlm.nih.gov/tools/primer-blast/) based on the sequences of the unigenes. The criteria for primer design were as follows: primer lengths of 17–24 bp, GC content of 50–66%, theoretical anneal at around 60 °C, and amplicon lengths of 100–200 bp. All primers were synthesized by the Beijing Genomics Institute (Beijing, China).

### qRT-PCR analysis

Sample reactions were performed in a 10 μL reaction volume with 5 μL of 2× qPCR MasterMix (#MasterMix-S, ABM, Canada), 1 μL cDNA template, 0.3 μL each primer, and 3.4 μL DNase/RNase-free water and run on the Bio-Rad CFX96 Real-Time PCR Detection System. The thermal cycling conditions were conducted according to the reagent kit instructions as follows: enzyme activation at 95 °C for 10 mins, followed by 40 cycles of denaturation at 95 °C for 15 s, annealing/extension at 60 °C for 60 s. The specificity of the SYBR green PCR signal was confirmed by melting curve analysis. All samples were analyzed in 3 replicates. Serial tenfold dilutions (dilution ratio 1:10^3^–1:10^10^) of cDNA template (note: in primer amplification test, cDNA template is PCR product with the same primer) were used to generate a slope of the standard curve to calculate the amplification efficiency and R^2^ of each paired primer. All qRT-PCR experiments and data analyses in the present study were performed following the MIQE guidelines [45, 46].

### Determination of expression stability of HKGs by four traditional algorithms

The Ct data of all the candidate HKGs obtained from the qRT-PCR experiments were evaluated by 4 algorithms, geNorm [26], NormFinder [28], BestKeeper [29], and the ΔCt method [27] (Fig. 7d).

### A comprehensive analysis was performed using a newly developed algorithm

After using the above-mentioned traditional evaluation algorithms, another algorithm was developed for comprehensive ranking. The new algorithm, ComprFinder, standardizes the output values from the above 4 algorithms then arithmetically averages them to get their FS and final ranking order (Fig. 7d).

**Fig. 8:**
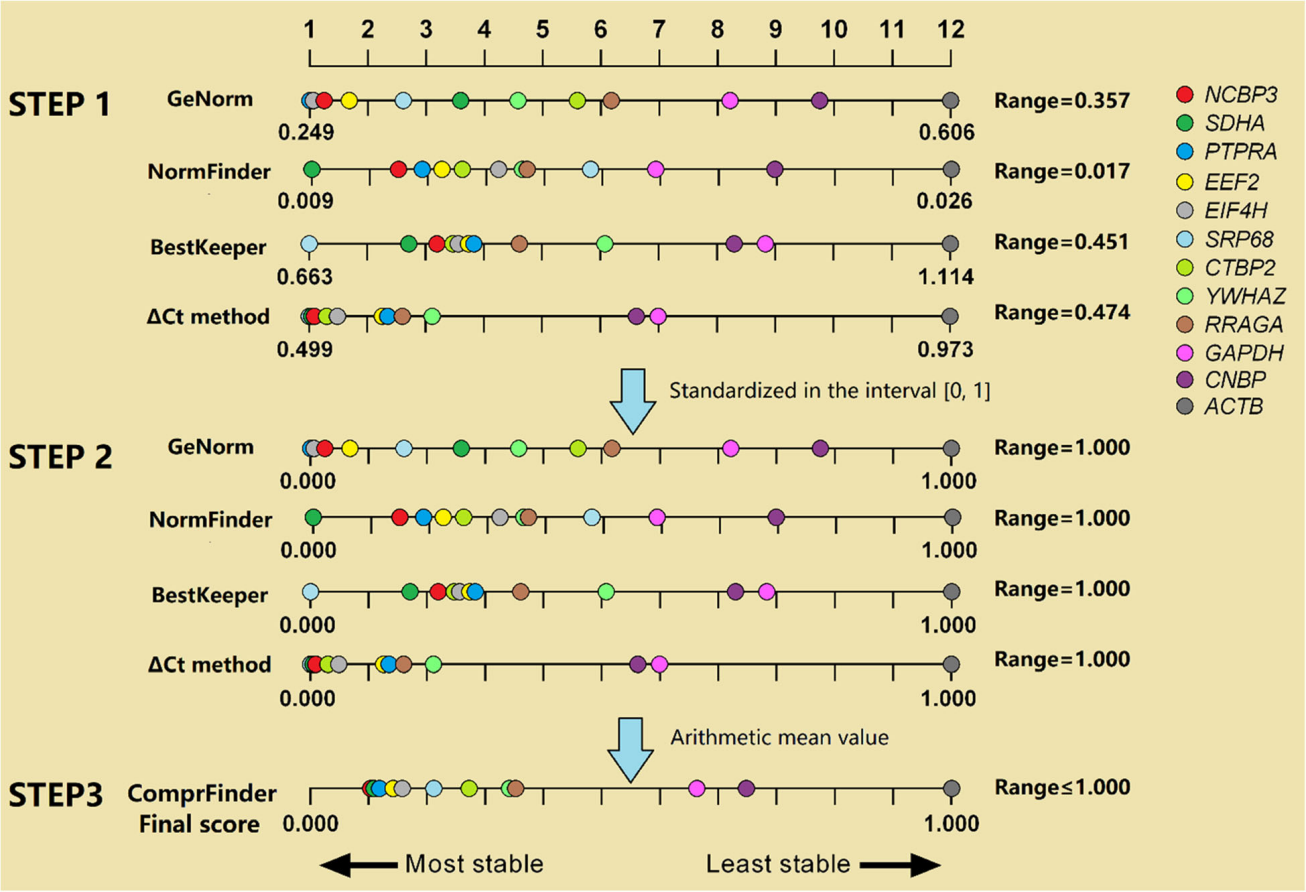
Schematic diagram of the ComprFinder algorithm. The same color presents the same gene. From step 1 to step 2, the uneven distribution of these 12 HKGs will not change, but they are proportionally enlarged or reduced to the range of 0–1. Calculated the arithmetic mean value for each gene to determine the final ranking order

The schematic diagram of the ComprFinder algorithm is presented in Fig. 8. Specifically, **STEP 1**, according to the original algorithm results ordered the values from small to large to find the minimum and the maximum. From this, the range was calculated (1) where A is one of the original algorithms, and is also used to calculate Δ_B_, Δ_C_, and Δ_D_. In **STEP 2**, the values from the original algorithm were standardized in the interval [0,1], where the minimum value = 0 and the maximum value = 1. The normalized value (A’_*i*_) was calculated (2) where *i* is one of these candidate HKGs, and is also used to calculate B’_*i*_, C’_*i*_, and, D’_*i*_. All the other data were assigned normalized values between 0 and 1. This step makes these stability values that belong to the same HKG but from different original algorithms abide by the additive property. In **STEP 3**, arithmetic averages of the standardized values for each candidate HKG were calculated, and then these FS were sorted to obtain their final rankings (Fig. 8). The FS_*i*_ (3) was determined with n being the number of original algorithms we used.

1 $$ {\Delta}_{\mathrm{A}}={\mathrm{A}}_{max}-{\mathrm{A}}_{min} $$

2 $$ \mathrm{A}{'}_i=\left({\mathrm{A}}_i-{\mathrm{A}}_{min}\right)/{\Delta}_{\mathrm{A}} $$

3 $$ \mathrm{F}{S}_i=\left(\mathrm{A}{'}_i+\mathrm{B}{'}_i+\mathrm{C}{'}_i+\mathrm{D}{'}_i\right)/\mathrm{n} $$

### Experimental validation of the HKGs

To verify the results, the 3 best candidate HKGs were selected (*NCBP3*, *SDHA*, and *PTPRA*), as well as the most unstable and most commonly used HKGs (*ACTB* and *GAPDH*). Next, the HKGs were verified and evaluated using 3 target genes (*DKK1*, *SHH*, and *FGF5*), which are the hot genes in hair follicle research (Fig. 7e). Considering the need to accurately evaluate target gene expression profile, 3 new bucks were added in the original sample size of 3 does, (6 adult IMCG, 3♂ and 3♀) and were sampled over 3 time-points (T1, T2, and T3) (Additional file 1: Table S6). The qRT-PCR was conducted as described in the determination stage. The paired sample *t*-test was performed using Microsoft Excel, and the graph was plotted using GraphPad Prism 6. The results are presented as Mean ± SEM, * *P* < 0.05, ** *P* < 0.01. For multiple gene combinations, the geometric average of their Ct value was calculated [26]. The relative gene expression level was calculated as 2^-ΔCt^, ΔCt = Δ (Ct_target gene_-Ct_HKGs_).

To further evaluate the internal relationship of these candidate HKGs, a correlation analysis was performed. First, the target genes were normalized by different HKGs or HKG combinations, to calculate their ΔCt values. Then their copy numbers were converted to relative expression levels with 2^-ΔCt^. Finally, their normalized-based expression levels were examined by the correlation analysis.

## Supplementary information

**Additional file 1: Figure S1.** The top 14 enriched signaling pathways of the 1325 candidate HKGs based on KEGG analysis. **Figure S2.** Melting curves for the 12 candidate HKGs and 3 target genes. **Figure S3.** Optimal number of HKGs in different experimental groups calculated by geNorm. Pairwise variation (V_n_/V_n + 1_) analysis between normalization factors (NF_n_ and NF_n + 1_) to calculate the number of HKGs required in each experimental condition (Groups 1–4, and all samples). **Table S1.** Primer sequences and amplicon information of candidate HKGs and target genes for qRT-PCR. **Table S2.** Ct values of the 12 candidate HKGs in all samples. **Table S3.** The comparison of experimental results and RNA-seq data. **Table S4.** The comparison of final results using ComprFinder and RefFinder algorithms. **Table S5.** The sample information in the determination stage. **Table S6.** The sample information in the validation stage.

**Additional file 2.** Downloadable Excel file with the ComprFinder algorithm.

**Additional file 3.** Matlab_script.

## Data Availability

The RNA-seq dataset analyzed during the current study is available in the Sequence Read Archive (SRA) database, accessible through https://www.ncbi.nlm.nih.gov/sra/?term=PRJNA630571.

## Abbreviations

ACTB: Actin Beta
CNBP: CCHC-Type Zinc Finger Nucleic Acid Binding Protein
Ct: Cycle threshold value
CTBP2: C-Terminal Binding Protein 2
CV: Coefficient of variation
DBG: Dazu black goat
DKK1: Dickkopf WNT Signaling Pathway Inhibitor 1
DPM: Dispersion measure
EEF2: Eukaryotic Translation Elongation Factor 2
EIF4H: Eukaryotic Translation Initiation Factor 4H
F1: First filial generation
FGF5: Fibroblast Growth Factor 5
FPKM: Fragments per kilobase of exon model per million mapped reads
FS: Final score
GAPDH: Glyceraldehyde-3-Phosphate Dehydrogenase
HCWG: Hechuan white goat
HKGs: House-keeping genes
IMCG: Inner Mongolia cashmere goat
MFC: Maximum fold change
NCBP3: Nuclear Cap Binding Subunit 3
PTPRA: Protein Tyrosine Phosphatase Receptor Type A
qRT-PCR: Quantitative real-time polymerase chain reaction
R^2^: Coefficient of determination
RNA-seq: RNA Sequencing
RRAGA: Ras Related GTP Binding A
SD: Standard deviation
SDHA: Succinate Dehydrogenase Complex Flavoprotein Subunit A
SHH: Sonic Hedgehog Signaling Molecule
SRP68: Signal Recognition Particle 68
YWHAZ: Tyrosine 3-Monooxygenase/Tryptophan 5-Monooxygenase Activation Protein Zeta
