# Supplementary material for: Transcriptome-based selection and validation of optimal house-keeping genes for skin research in goats (Capra hircus)
Source: BMC Genomics. 2020 Jul 18;21:493. doi: 10.1186/s12864-020-06912-4 (PMC7368715; doi:10.1186/s12864-020-06912-4)
Supplement: Supplementary file 1 — Additional file 1: Figure S1. The top 14 enriched signaling pathways of the 1325 candidate HKGs based on KEGG analysis. Figure S2. Melting curves for the 12 candidate HKGs and 3 target genes. Figure S3. Optimal number of HKGs in different experimental groups calculated by geNorm. Pairwise variation (Vn/Vn + 1) analysis between normalization factors (NFn and NFn + 1) to calculate the number of HKGs required in each experimental condition (Groups 1–4, and all samples). Table S1. Primer sequences and amplicon information of candidate HKGs and target genes for qRT-PCR. Table S2. Ct values of the 12 candidate HKGs in all samples. Table S3. The comparison of experimental results and RNA-seq data. Table S4. The comparison of final results using ComprFinder and RefFinder algorithms. Table S5. The sample information in the determination stage. Table S6. The sample information in the validation stage. [file 12864_2020_6912_MOESM1_ESM.pdf]

Supplementary Figures and Tables for

Transcriptome-based Selection and Validation of Optimal House-keeping Genes for Skin Research in Goats (*Capra hircus*)

Jipan Zhang, Chengchen Deng, Jialu Li, Yongju Zhao

Yongju Zhao
E-mail: zyongju@163.com

This PDF file includes:

Figures S1-S3
Tables S1-S6
References for supplementary Figures and Tables

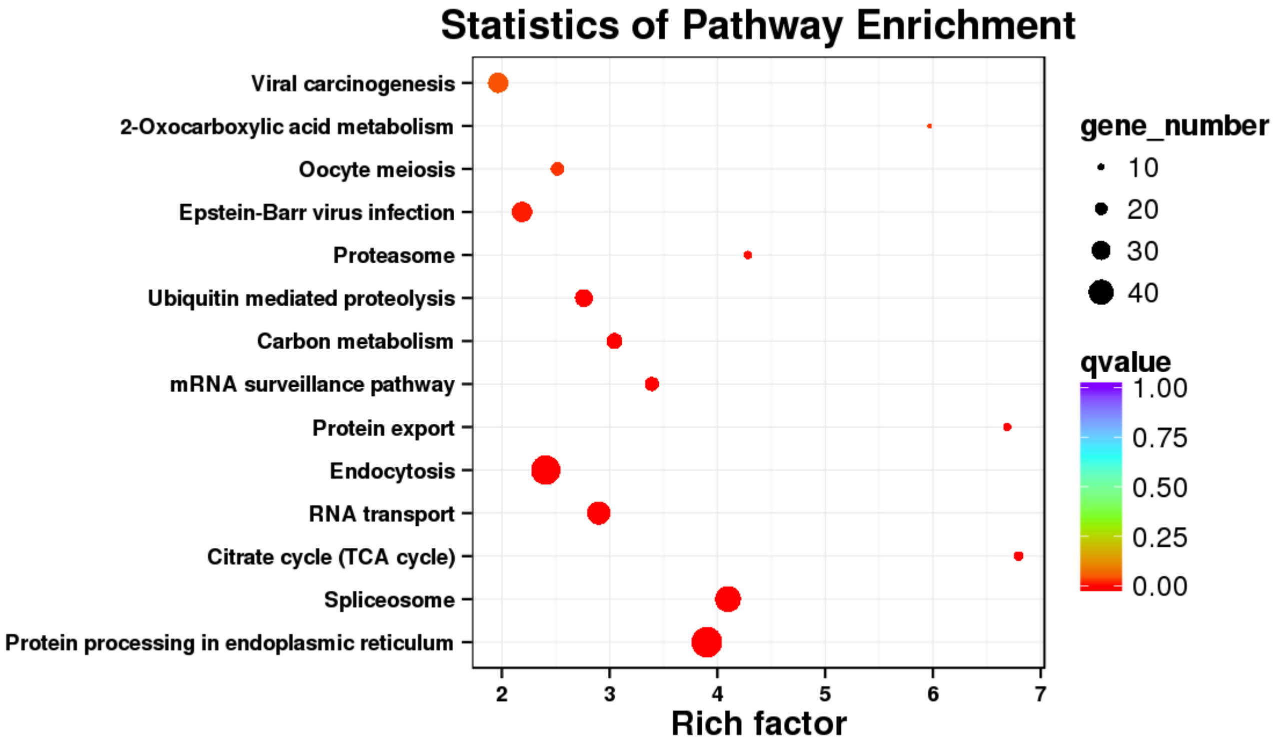

Figure S1 The top 14 enriched signaling pathways of the 1325 candidate HKGs based on KEGG analysis

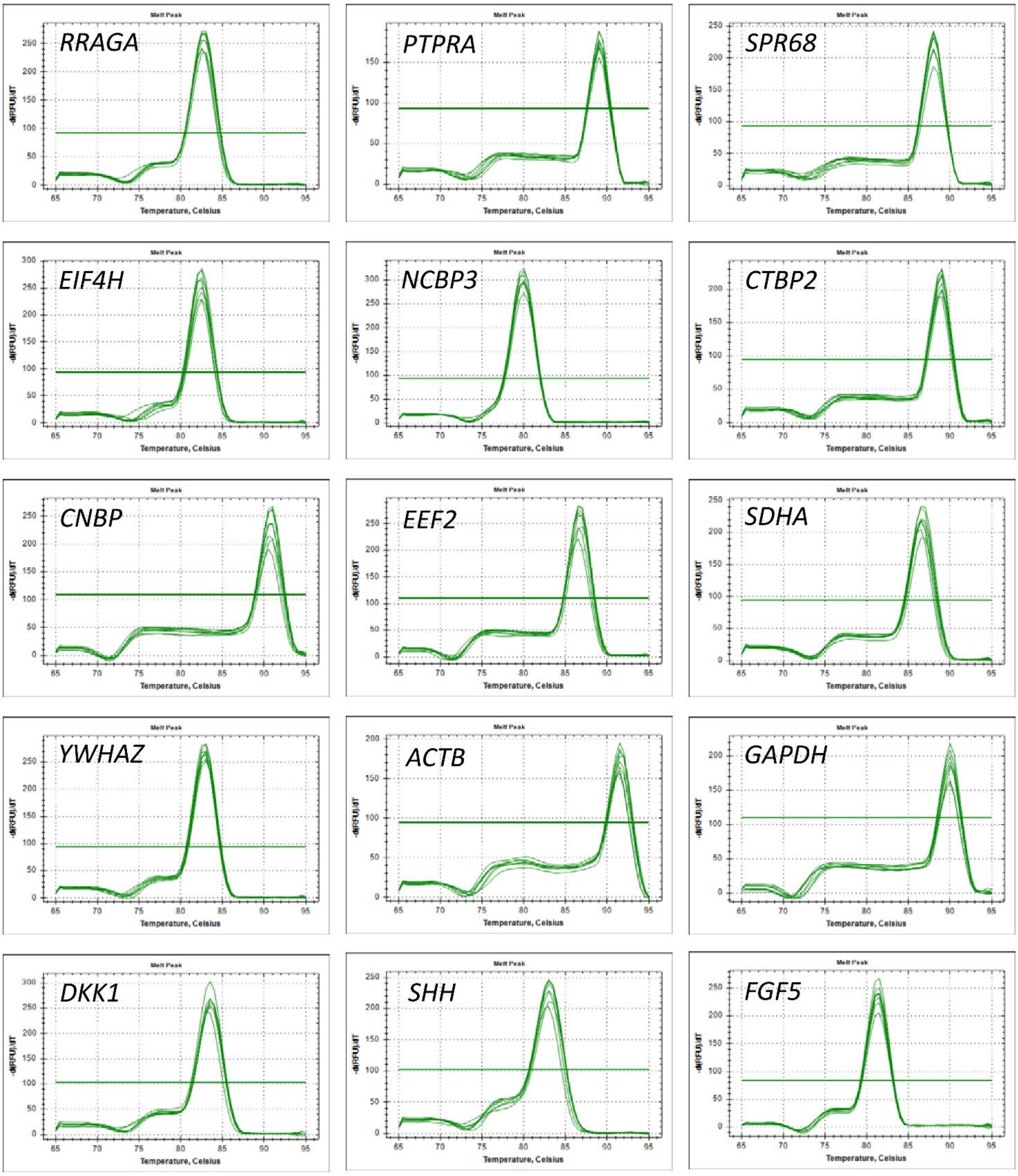

Figure S2 Melting curves for the 12 candidate HKGs and 3 target genes

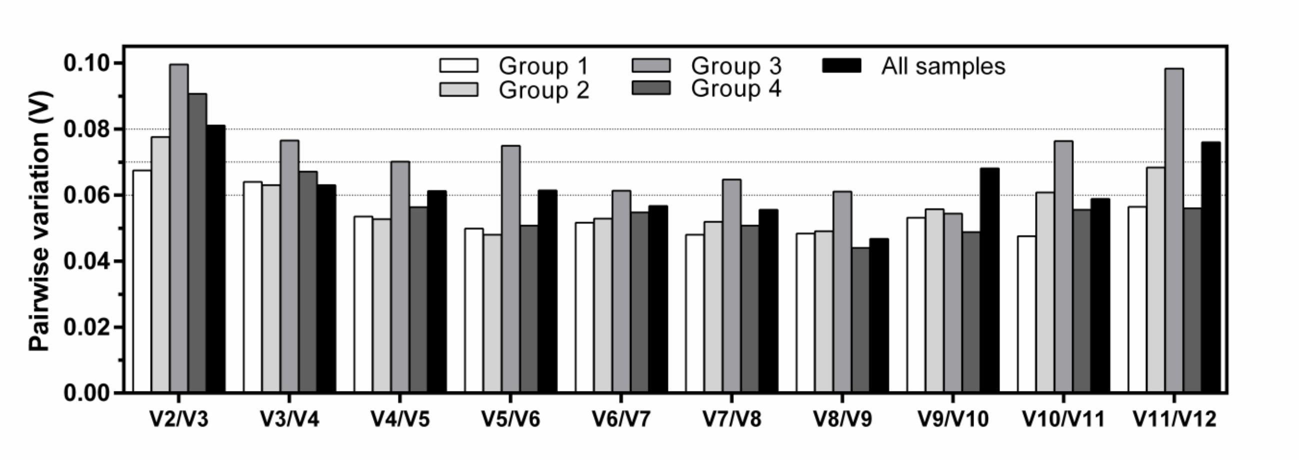

Figure S3 Optimal number of HKGs in different experimental groups calculated by geNorm
Pairwise variation (V_n/V_{n+1}) analysis between normalization factors (NF_n and NF_{n+1}) to calculate the number of HKGs required in each experimental condition (Groups 1-4, and all samples)

Table S1 Primer sequences and amplicon information of candidate HKGs and target genes for qRT-PCR

Gene symbol	Description	NCBI Gene ID	Forward primer/ Reverse primer	Theoretical Tm (°C) [a]	Product size (bp)	Primer efficiency (%) [b]	Coefficient of determination (R^2) [c]
RRAGA	Ras Related GTP Binding A	102185564	ATCCCTTCTGCGGCTACTCT<br>TCAACGCATAAGGAGGCTGT	60.40<br>59.39	102	100.7%	0.9995
PTPRA	Protein Tyrosine Phosphatase Receptor Type A	102184258	AATTCAACGCTCTCCCTGCT<br>AACTGGTGTCAGATGGACTCG	59.67<br>59.73	131	103.9%	0.9996
SRP68	Signal Recognition Particle 68	102188706	ATAAGGAAAACGAGCGGCCT<br>TGTCTACGCGAACAGTAGCC	59.75<br>59.83	160	102.7%	0.9998
EIF4H	Eukaryotic Translation Initiation Factor 4H	102177396	TCAGCATAAGGAGTGTACGGC<br>ATGTCCACACGAAGTGACCG	59.86<br>60.32	151	103.0%	0.9999
NCBP3	Nuclear Cap Binding Subunit 3	102180078	AGGAACTCCATGAGGGCAGA<br>GACGTGTGTGCTGACGTTTT	60.25<br>59.63	127	101.9%	0.9998
CTBP2	C-Terminal Binding Protein 2	102173064	TGCCACATACAGATACCCGC<br>TTGGGGTGCTCTCGATTGTC	59.89<br>60.04	183	101.5%	0.9993
CNBP	CCHC-Type Zinc Finger Nucleic Acid Binding Protein	102168217	CATCGCCAAGGACTGCAAGG<br>TACGTGACCAGTTTCGCCAC	61.65<br>60.60	193	99.6%	0.9994
EEF2	Eukaryotic Translation Elongation Factor 2	102176034	TCTTCTCAGGGCTGGTGTCT<br>CCACAAGGCACATCCTCGAT	60.18<br>60.11	154	104.3%	0.9999
SDHA	Succinate Dehydrogenase Complex Flavoprotein Subunit A	102183107	CGCTACGACACCAGCTACTT<br>TGGACCCGTCTTCTATGCAC	59.83<br>59.46	103	102.3%	0.9998
YWHAZ	Tyrosine 3-Monooxygenase/Tryptophan 5-Monooxygenase Activation Protein Zeta	102175223	TCGCTACTTGGCTGAGGTTG<br>AGTTAAGGGCCAGACCCAGT	60.04<br>60.48	143	101.1%	0.9998
ACTB	Actin Beta	102179831	TGATATTGCTGCGCTCGTGGT<br>GTCAGGATGCCTCTCTTGCTC	62.76<br>60.47	189	102.2%	0.9986
GAPDH	Glyceraldehyde-3-Phosphate Dehydrogenase	100860872	TTATGACCACTGTCCACGCC<br>TCAGATCCACAACGGACACG	60.04<br>60.04	216	97.0%	0.9995
DKK1	Dickkopf WNT Signaling Pathway Inhibitor 1	102175223	AGGTCAAGTGTGCACCAAGC<br>GTGATCTTTCTGCATCCGGC	61.10<br>59.34	106	96.4%	0.9995
SHH	Sonic Hedgehog Signaling Molecule	102170201	CCCCAATTACAACCCCGACA<br>ATGGCTAAGGCGTTCAGCTT	59.96<br>60.04	102	97.4%	0.9991
FGF5	Fibroblast Growth Factor 5	102171226	TTGCTGTGTCTCAGGGGATTG<br>AGTTCTGTGTATGGCGGAGG	60.27<br>59.96	173	99.6%	0.9995

[a] Theoretical Tm (°C) was calculated by NCBI website

[b] The primer amplification efficiency was determined between 95.0%~105.0%

[c] The coefficient of determination (R^2) was determined > 0.9900

Table S2 Ct values of the 12 candidate HKGs

All levels	Biological replicates	*NCBP3*	*SRP68*	*RRAGA*	*EIF4H*	*CTBP2*	*PTPRA*	*CNBP*	*EEF2*	*SDHA*	*YWHAZ*	*ACTB*	*GAPDH*
IMCG_T1	1	28.16434	28.70550	24.34091	24.96209	26.55112	26.80482	23.55182	23.44156	27.52744	23.68742	22.39479	26.41359
	2	28.25713	29.32586	23.75713	24.96831	26.97258	26.97641	22.77509	23.18404	27.21869	23.44786	22.40595	25.57133
	3	27.91557	29.33885	23.91899	24.52127	26.20905	26.60869	23.33491	22.99505	27.44909	23.53368	23.54350	25.97279
IMCG_T2	1	29.28326	29.13997	25.00386	25.49840	27.51619	27.41542	25.55930	24.23757	28.04912	24.18984	24.10591	28.00869
	2	27.61504	28.34770	23.31686	23.91585	26.43132	25.75075	23.92695	22.88517	27.12907	22.41540	23.67703	25.82418
	3	27.84793	28.61623	23.16576	24.35848	25.81903	26.07078	23.48709	23.16417	26.94849	22.04147	23.37197	25.38546
IMCG_T3	1	26.96265	27.45348	22.97701	23.31623	25.44461	25.17183	22.85815	22.01177	25.84413	21.52672	21.97679	24.51584
	2	26.70307	28.00336	23.02843	23.44450	25.80837	25.29400	22.92014	22.08848	26.46017	22.36284	22.74948	25.30336
	3	27.76183	29.13608	23.42081	24.64794	25.61177	26.24652	22.86922	22.80294	26.88725	23.09005	24.08174	25.17775
DBG_T1	1	28.80822	29.68228	25.26935	25.51322	27.72832	27.55854	24.60033	24.10725	28.43124	24.69280	23.20744	27.23577
	2	28.62290	29.52190	25.09195	25.47243	27.53072	27.26127	24.29982	23.59859	28.04012	24.38131	23.40778	26.82453
	3	28.25096	29.27698	24.77324	24.97822	27.73896	26.95808	25.45079	23.43989	28.04844	24.01048	25.04039	27.29102
HCWG	1	29.83407	30.23241	24.90798	26.16283	27.96830	28.25143	24.03682	24.97582	28.76931	25.38844	24.73200	26.60693
	2	30.71078	30.75833	26.30710	26.88776	29.41073	29.05004	28.34459	26.15881	30.11983	26.35229	28.81975	30.00812
	3	27.30543	28.04657	22.88480	23.72016	26.61259	25.34976	23.35408	22.90262	27.19157	22.25539	24.08277	25.47276
F_1_Adult	1	30.02821	30.92856	24.46928	26.40394	27.48942	28.01023	23.98699	25.00404	28.64008	24.49915	24.31338	27.01407
	2	28.50506	28.97286	22.69740	24.81879	25.72160	26.28651	22.53236	23.23632	26.67668	22.80372	22.72530	25.28433
	3	28.37174	28.82229	23.74817	24.43774	26.48737	26.28721	24.04480	23.05613	27.13610	23.37151	22.77002	26.34717
F_1_P0	1	27.58018	28.97954	23.15243	24.27326	25.70100	26.34427	22.26956	22.79725	26.66832	23.02963	21.70964	24.43398
	2	28.35736	29.13490	24.39972	25.20921	26.54810	27.23559	23.53062	24.06635	27.59964	23.68766	22.63967	26.69971
	3	27.20285	28.11182	22.76539	23.55574	25.34211	25.56703	21.92613	22.17345	26.02789	22.21240	20.73795	24.51585
F_1_P60	1	27.64457	28.72744	23.70623	24.26161	25.67872	26.14214	22.90989	22.62088	26.99135	23.25107	21.50003	25.59679
	2	28.26994	29.34304	24.49751	24.94233	26.62829	26.76872	23.63084	23.27155	27.06863	23.87321	21.90457	25.87574
	3	27.99638	29.19163	24.61050	24.78190	26.53662	26.54169	23.92446	23.19048	27.43699	23.69908	22.20007	26.48228
F_1_P240	1	27.37825	28.27204	23.21795	24.22030	26.27306	25.77934	22.42973	22.90259	26.68181	22.34999	21.55132	24.55379
	2	27.41644	28.96612	23.22677	24.49185	26.30158	26.13525	22.14795	23.01898	26.57169	22.61358	22.46594	23.50751
	3	29.01975	29.68124	25.22753	25.63810	27.76522	27.58678	24.42935	24.13927	27.84051	24.16605	23.32316	26.84842
IMCG #4	1	27.12446	28.21862	23.15035	23.83500	25.75247	25.50506	22.27406	22.02559	26.56386	21.87257	21.82809	24.98706
	2	30.62798	31.64029	26.58511	28.04203	28.69413	29.02578	25.60342	26.07496	30.44668	25.64969	25.71211	28.09351
	3	28.61493	29.52551	23.75789	25.12668	26.58596	26.71837	23.38992	23.46683	28.02151	23.13910	24.25547	25.61146
IMCG #5	1	27.47526	28.34281	23.06095	24.04642	25.92586	25.70303	22.65681	22.32825	27.19915	22.47791	22.67128	25.14689
	2	29.10099	30.07991	24.03633	25.56652	26.79606	27.56781	24.29628	23.75707	27.87092	24.23309	24.84449	25.82487
	3	27.83382	28.44570	24.22073	24.14061	26.51585	26.21232	24.54648	23.01782	27.35720	23.22623	23.30217	27.08007
IMCG #12	1	28.04761	28.57298	23.10843	24.23510	26.21650	26.60600	23.15357	23.00377	26.85139	22.61188	22.82622	25.34926
	2	27.08271	28.03408	23.33371	23.56213	26.09230	25.50049	23.12859	22.19729	26.79689	22.03642	21.95983	25.79002
	3	28.54629	29.06722	24.01722	25.04703	26.76913	27.04596	24.81492	23.83957	27.50276	23.06568	24.15611	26.80194
IMCG #14	1	29.20335	30.00103	25.78394	25.81831	28.84107	27.58951	26.13512	24.73568	28.61704	24.85975	24.92782	28.28207
	2	27.70444	28.33221	23.48891	23.99042	26.16592	25.79009	23.18880	22.61617	27.10429	23.03411	22.41923	24.83131
	3	29.37377	29.85678	24.84813	25.79908	27.68507	27.44464	24.97184	24.76488	28.79529	24.16867	25.04682	26.49276

Table S3 The comparison of experimental results and RNA-seq data

Gene	FPKM	Type	Experiment result		RNA-seq	
			Final Score	Final Ranking order	The CV (%)	Order by CV
NCBP3	10.0	New predicted candidate HKG	0.096	1	9.5%	5
SDHA	44.0	Suggested by previous study	0.099	2	18.5%	9
PTPRA	23.8	New predicted candidate HKG	0.108	3	9.1%	2
EEF2	499.7	New predicted candidate HKG	0.129	4	15.1%	8
EIF4H	133.0	New predicted candidate HKG	0.143	5	9.5%	4
SRP68	27.2	New predicted candidate HKG	0.192	6	9.2%	3
CTBP2	22.5	New predicted candidate HKG	0.248	7	9.9%	6
YWHAZ	137.5	Suggested by previous study	0.311	8	19.2%	10
RRAGA	51.4	New predicted candidate HKG	0.320	9	8.4%	1
GAPDH	391.6	Conventional HKG	0.603	10	29.9%	12
CNBP	226.5	New predicted candidate HKG	0.680	11	14.3%	7
ACTB	556.1	Conventional HKG	1.000	12	24.6%	11

Table S4 The comparison of final results using ComprFinder and RefFinder algorithms

Ranking order	Group 1		Group 2		Group 3		Group 4		All samples	
	ComprFinder	RefFinder	ComprFinder	RefFinder	ComprFinder	RefFinder	ComprFinder	RefFinder	ComprFinder	RefFinder
1	*EIF4H*(0.063)	*SDHA*(2.00)	*SDHA*(0.059)	*SDHA*(1.78)	*SDHA*(0.129)	*SDHA*(2.06)	*NCBP3*(0.105)	*NCBP3*(1.86)	*NCBP3*(0.096)	*PTPRA*(2.14)
2	*PTPRA*(0.090)	*PTPRA*(2.45)	*NCBP3*(0.082)	*EEF2*(2.00)	*PTPRA*(0.170)	*PTPRA*(2.66)	*PTPRA*(0.105)	*EEF2*(2.55)	*SDHA*(0.099)	*SDHA*(2.45)
3	*SDHA*(0.093)	*EIF4H*(2.45)	*EEF2*(0.090)	*NCBP3*(3.22)	*EIF4H*(0.180)	*EIF4H*(2.78)	*EEF2*(0.193)	*PTPRA*(2.59)	*PTPRA*(0.108)	*EEF2*(3.13)
4	*EEF2*(0.171)	*EEF2*(3.16)	*EIF4H*(0.210)	*EIF4H*(3.64)	*SRP68*(0.230)	*EEF2*(4.43)	*SDHA*(0.211)	*SDHA*(4.28)	*EEF2*(0.129)	*NCBP3*(3.46)
5	*SRP68*(0.174)	*SRP68*(3.96)	*RRAGA*(0.227)	*PTPRA*(3.83)	*EEF2*(0.247)	*YWHAZ*(4.53)	*SRP68*(0.245)	*CTBP2(4.56)*	*EIF4H*(0.143)	*EIF4H*(3.5)
6	*YWHAZ*(0.256)	*YWHAZ*(5.60)	*PTPRA*(0.236)	*RRAGA*(5.18)	*NCBP3*(0.252)	*SRP68*(4.73)	*CTBP2*(0.263)	*RRAGA*(5.03)	*SRP68*(0.192)	*SRP68*(4.49)
7	*NCBP3*(0.282)	*CTBP2*(6.26)	*CTBP2*(0.322)	*CTBP2*(6.74)	*YWHAZ*(0.277)	*NCBP3*(5.24)	*EIF4H*(0.309)	*SRP68*(5.9)	*CTBP2*(0.248)	*CTBP2*(5.73)
8	*CTBP2*(0.358)	*NCBP3*(6.45)	*SRP68*(0.430)	*SRP68*(6.84)	*CTBP2*(0.293)	*CTBP2*(5.83)	*RRAGA*(0.327)	*EIF4H*(6.45)	*YWHAZ*(0.311)	*YWHAZ*(7.45)
9	*RRAGA*(0.605)	*RRAGA*(9.74)	*GAPDH*(0.609)	*YWHAZ*(8.85)	*GAPDH*(0.399)	*RRAGA*(7.97)	*YWHAZ*(0.361)	*YWHAZ*(6.65)	*RRAGA*(0.320)	*RRAGA*(8.00)
10	*CNBP*(0.637)	*CNBP*(9.95)	*YWHAZ*(0.630)	*GAPDH*(10.00)	*RRAGA*(0.404)	*GAPDH*(8.68)	*ACTB*(0.795)	*ACTB*(10.00)	*GAPDH*(0.603)	*GAPDH*(10.24)
11	*ACTB*(0.677)	*ACTB*(10.22)	*CNBP*(0.697)	*CNBP*(10.16)	*CNBP*(0.730)	*CNBP*(11.00)	*CNBP*(0.820)	*CNBP*(11.24)	*CNBP*(0.680)	*CNBP*(10.74)
12	*GAPDH*(0.971)	*GAPDH*(12.00)	*ACTB*(0.943)	*ACTB*(11.74)	*ACTB*(1.000)	*ACTB*(12.00)	*GAPDH*(0.994)	*GAPDH*(11.74)	*ACTB*(1.000)	*ACTB*(12.00)

Note: the RefFinder results were calculated at https://www.heartcure.com.au/reffinder/?type=reference, accessed 24 Feb 2020.

Table S5 The sample information in the determination stage

<table>
<thead>
<tr><th>Levels</th><th>Biological replicates</th><th>Group 1</th><th>Group 2</th><th>Group 3</th><th>Group 4</th><th>All samples</th><th>Animal source</th><th>Sampling procedure</th><th>Sample preserved in time</th><th>Sample long-term preservation</th><th>Note</th></tr>
</thead>
<tbody>
<tr><td>IMCG_T1</td><td>1
2
3</td><td>√</td><td></td><td>√</td><td></td><td>√</td><td rowspan="9">Breeding farm of Southwest University, Chongqing, China</td><td rowspan="9">This procedure was referred to previous similar studies [1] and was performed as follows.
(1) Skin fibers on the lateral chest were first shed by an electric hair clipper (FC5902, Flyco, China) and then removed by a lint remover (SR7811, Superman, China) with a 5 cm × 5 cm area.
(2) Animals were anesthetized by intramuscular injection of xylazine hydrochloride (#180121777, Baite, Changsha, China), according to 0.2 mg per kg body weight.
(3) Animals were transferred to an operating table and the exposed skin was swabbed with alcohol to disinfect.
(4) Each skin tissue approximately 1 cm^2 was grasped with sterile forceps and quickly cut near the tip using sterile scalpel blades.
(5) The surgical site was closed using a suture, and sterilized with iodophor.
(6) Injection of idazoxan hydrochloride (#180122315, Baite, Changsha, China), according to 0.2 mg per kg body weight.
(7) After the surgical procedure, animals were released and returned to the pen.</td><td rowspan="9">Liquid nitrogen</td><td rowspan="9">−80 °C</td><td rowspan="9">a</td></tr>
<tr><td>IMCG_T2</td><td>1
2
3</td><td>√</td><td></td><td></td><td>√</td><td>√</td></tr>
<tr><td>IMCG_T3</td><td>1
2
3</td><td>√</td><td></td><td></td><td></td><td>√</td></tr>
<tr><td>DBG_T1</td><td>1
2
3</td><td></td><td></td><td>√</td><td></td><td>√</td></tr>
<tr><td>HCWG</td><td>1
2
3</td><td></td><td></td><td>√</td><td></td><td>√</td></tr>
<tr><td>F_1_Adult</td><td>1
2
3</td><td></td><td>√</td><td>√</td><td></td><td>√</td></tr>
<tr><td>F_1_P0</td><td>1
2
3</td><td></td><td>√</td><td></td><td></td><td>√</td></tr>
<tr><td>F_1_P60</td><td>1
2
3</td><td></td><td>√</td><td></td><td></td><td>√</td></tr>
<tr><td>F_1_P240</td><td>1
2
3</td><td></td><td>√</td><td></td><td></td><td>√</td></tr>
<tr><td>IMCG #4</td><td>1
2
3</td><td></td><td></td><td></td><td>√</td><td>√</td><td rowspan="4">Private farms, in the Inner Mongolia Autonomous Region, China</td><td rowspan="4">This procedure was described in our previous publication [2] and was performed as follows.
(1) Skin fibers on the different points we designed were shed and removed as above mentioned.
(2) After using the electric shock, goats were performed the commercial slaughter then followed by jugular vein bloodletting method.
(3) Immediately after animal sacrifice, the skin samples were taken from different body sites (#4, #5, #12, and #14) as quickly as possible.</td><td rowspan="4">RNA/DNA sample protection reagent (Takara, Dalian, China)</td><td rowspan="4">−80 °C</td><td rowspan="4">b</td></tr>
<tr><td>IMCG #5</td><td>1
2
3</td><td></td><td></td><td></td><td>√</td><td>√</td></tr>
<tr><td>IMCG #12</td><td>1
2
3</td><td></td><td></td><td></td><td>√</td><td>√</td></tr>
<tr><td>IMCG #14</td><td>1
2
3</td><td></td><td></td><td></td><td>√</td><td>√</td></tr>
</tbody>
</table>

a. these goats were well cared for and were recovered in two weeks after surgery.

b. a goat can provide #4, #5, #12, and #14 samples, so only 3 goats were sacrificed in this study; the procedure step (2) was executed by the slaughter company (Jiaerba, Baotou city); after sampling, the animal carcass was further processed in the slaughterhouse, because it was not contaminated by our sampling procedure and the anesthesia was not used.

Table S6 The sample information in the validation stage

Levels	Biological replicates	Description
IMCG_T1	1	Come from the determination stage
	2	
	3	
	4	New samples for the requirement of accurately evaluate the expression
	5	
	6	
IMCG_T2	1	Come from the determination stage
	2	
	3	
	4	New samples for the requirement of accurately evaluate the expression
	5	
	6	
IMCG_T3	1	Come from the determination stage
	2	
	3	
	4	New samples for the requirement of accurately evaluate the expression
	5	
	6	

Note: the samples 4-6 were collected as samples 1-3 at the same time and the same place, and the same procedure was performed.

References

1. Rile N, Liu Z, Gao L, Qi J, Zhao M, Xie Y, Su R, Zhang Y, Wang R, Li J *et al*: **Expression of Vimentin in hair follicle growth cycle of inner Mongolian Cashmere goats**. *Bmc Genomics* 2018, **19**:1-8.

2. Zhang JP, Deng CC, Chen SR, Zhao L, Zhao YJ: **Effect of body site on hair follicle density in Inner Mongolia Cashmere goat (*Capra hircus*)**. *Small Ruminant Res* 2020, **191**:1-6.
